# Rapid Estimation of Binding Constants for Cucurbit[8]uril Ternary Complexes Using Electrochemistry

**DOI:** 10.1021/acs.analchem.0c04887

**Published:** 2021-02-17

**Authors:** Jia Liu, Hugues Lambert, Yong-Wei Zhang, Tung-Chun Lee

**Affiliations:** † Institute for Materials Discovery, University College London (UCL), Bloomsbury, London WC1E 7JE, United Kingdom; ‡ Department of Chemistry, University College London (UCL), 20 Gordon Street, London, WC1H 0AJ, United Kingdom; § Institute of High Performance Computing, 1 Fusionopolis Way, 138632, Singapore

## Abstract

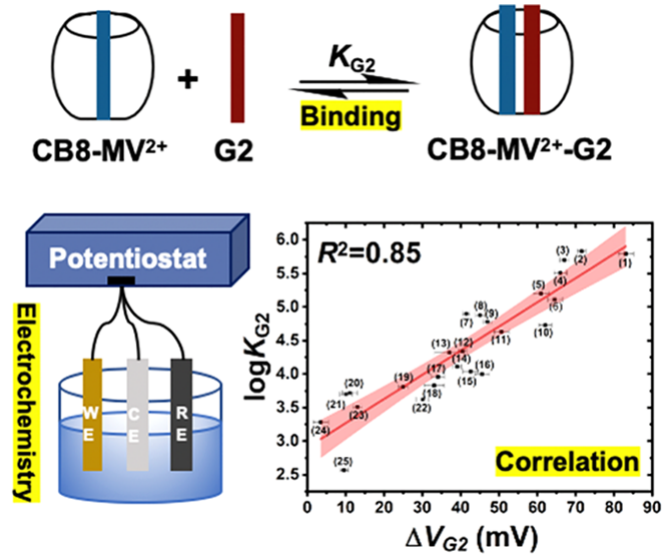

Supramolecular complexes are of fundamental interests in biomedicines and adaptive materials, and thus facile methods to determine their binding affinity show usefulness in the design of novel drugs and materials. Herein, we report a novel approach to estimate the binding constants *K*_G2_ of cucurbit[8]uril-methyl viologen-based ternary complexes (CB8-MV^2+^-G2) using electrochemistry, achieving high precision (±0.03) and practical accuracy (±0.32) in log*K*_G2_ and short measurement time (<10 min). In particular, we have uncovered a linear correlation (*R*^2^ > 0.8) between the reduction potential of CB8-MV^2+^-G2 ternary complexes and their reported binding constants from isothermal titration calorimetry, which allow a calibration curve to be plotted based on 25 sample complexes. Mechanistic investigation using experimental and computational approaches reveals that this correlation stems from the dynamic host-guest exchange events occurring after the electron transfer step. Binding constants of unknown ternary complexes, where G2 = hydrocarbons, were estimated, illustrating potential applications for sparsely soluble second guests.

Quantitative study of noncovalent interactions between host and guest molecules can offer crucial information to tailor the design of supramolecular materials for different applications, such as self-assembly of functional nanomaterials,^1−7^ catalysis and reaction modulation,^8−12^ selective sensing of small molecules,^13−16^ and triggered drug delivery and activation.^17−20^ Binding constants have been regarded as a basic criterion for assessing the binding strength of supramolecular complexes.^21^ The most common approaches for determining the binding constants are titration methods, in which the physical properties such as heat transfer (as measured by isothermal titration calorimetry, ITC), UV–vis absorption, chemical shift (as measured by nuclear magnetic resonance, NMR), and fluorescence of a system are tracked during gradual addition of a guest into the solution of a host or vice versa.^21^ ITC is considered to show the most widespread applicability among these titration methods, yet it still suffers from inherent limitations of titration techniques, which include multiple measurement steps, complex data analysis, and limited applicability toward sparsely soluble guests. Aside from titration methodologies, surface plasmon resonance (SPR) and quartz crystal microbalance (QCM) are substrate-based techniques that can be utilized to kinetically investigate the binding strength of molecular interactions in a high-throughput fashion. However, their intrinsic limits of detection only allow measurement of species with a large molecular weight, for example, protein and antibodies.^22,23^

Cucurbit[*n*]urils (CB*n*, *n* = 5–8) are macrocyclic molecules that possess well-defined and highly hydrophobic cavities, and carbonyl-lined electron-rich portals.^17,24,25^ By taking advantage of the ion-dipole interactions with the portals and hydrophobic effect of the cavity of CBs, guests can bind to CB hosts to form inclusion complexes with high binding affinities in aqueous media. The millimolar solubility in water of CB complexes allows convenient electrochemical measurements which require electrically conducting media.

Compared to other CB homologues, CB8 is unique due to its larger cavity volume (367 Å^3^) and its ability to simultaneously encapsulate up to two guests to form either homo- or heteroternary complexes.^25−30^ For instance, CB8 can encapsulate two π-conjugated molecules and facilitate their heterodimerization within its cavity.^31^ In particular when electron-deficient dicationic methyl viologen (MV^2+^) serves as the first guest, a wide range of aromatic molecules can readily act as second guests (G2) to form 1:1:1 ternary complexes as CB8-MV^2+^-G2 with hydrophobic effects and charge-transfer interactions as driving forces of the complexation.^27,28,32−34^ Interestingly, the reduction potential of MV^2+^ can be altered upon formation of host-guest complexes with CB7 and CB8,^34−36^ owing to electrostatic stabilization and the formation of 1:2 complexes, respectively. Hence, MV^2+^ can potentially be exploited as a redox probe for studying host-guest complexation. Nevertheless, quantitative correlation between the supramolecular properties of a series of G2 and their electrochemical measurables has not been uncovered so far.

Herein, we report a simple and high-throughput scheme for estimating the binding constants of CB8-MV^2+^-G2 ternary complexes (*K*_G2_ in Figure 1a) using electrochemical techniques. The accuracy of log*K*_G2_ determined by this approach is approximated to be within a practical range of ±0.32, and the precision is estimated to be ±0.03. The entire measurement process can be completed within 10 min, which is considerably faster than traditional titration methods (e.g., ∼5 h for ITC). In particular, 25 CB8-MV^2+^-G2 ternary complexes were chosen as reference analytes, whose binding constants have already been determined using ITC.^32^ We measured the reduction potentials of these 25 ternary complexes as well as the CB8-MV^2+^ binary complex using cyclic voltammetry (CV) and square wave voltammetry (SWV), and then the shifts of reduction potential of each ternary complex with respect to that of CB8-MV^2+^ (Δ*V*_G2_ in Figure 1c) were extracted. Notably, a linear correlation (*R*^2^ > 0.8) between potential shifts of ternary complexes Δ*V*_G2_ and their ITC binding constants log*K*_G2_ was demonstrated. Mechanistic investigations using experimental and computational approaches reveal that this correlation stems from the dynamic host-guest exchange events occurring after the electron transfer step. As a proof-of-concept application, the log*K*_G2_ of CB8-MV^2+^-G2 ternary complexes, where G2 = a series of cyclic hydrocarbons, were estimated by using the linear regression result, illustrating direct detection of association strength of sparsely miscible/soluble and volatile compounds in aqueous environment which cannot be achieved otherwise.

**Figure 1:**
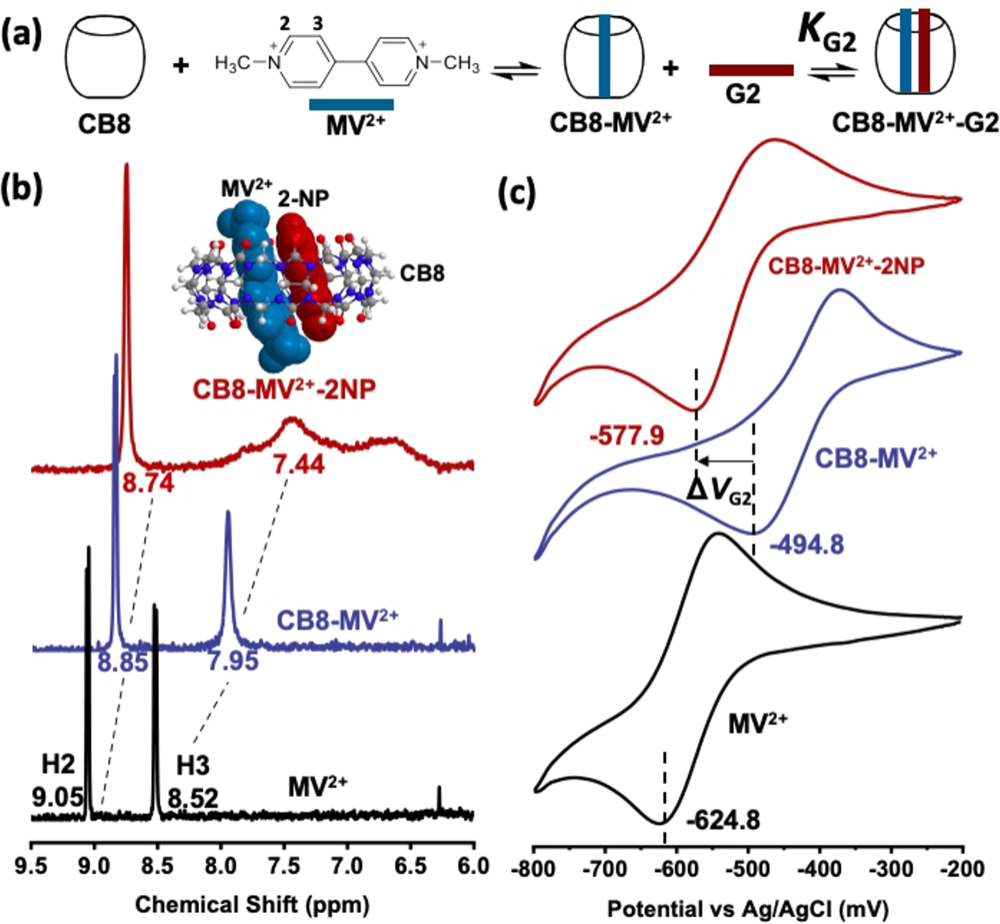
(a) Sequential complexation process of CB8 with MV^2+^ and 2NP as G2, where *K*_G2_ is the binding constant of the second equilibrium. (b) ^1^H NMR spectra (Inset: energy-minimized molecular model of a CB8-MV^2+^-2NP ternary complex at CPCM/wB97XD/6-31G* level of theory) and (c) cyclic voltammogram of free MV^2+^, 1:1 CB8-MV^2+^ and 1:1:1 CB8-MV^2+^-2NP ternary complex on a gold disk electrode (0.0314 cm^2^) vs Ag/AgCl. Medium: 6.25 mM phosphate buffer solution (pH 7.0). Scan rate: 10 mV/s. Δ*V*_G2_ = | *E*_CB8-MV-G2_ – *E*_CB8-MV_ |, where *E*_CB8-MV-G2_ and *E*_CB8-MV_ are the reduction potential of the CB8-MV^2+^-G2 ternary complex and the CB8-MV^2+^ binary complex, respectively.

## Experimental Methods

### Materials

Except 1,7-dihydroxynaphthalene, all other 24 guest molecules, methyl viologen dichloride hydrate (98%) and sodium phosphate monobasic were purchased from Sigma-Aldrich. 1,7-dihydroxynaphthalene and sodium phosphate dibasic heptahydrate were bought from Alfa Aesar and Fisher Scientific, respectively. All chemicals ordered were of analytical grade and used directly as received without any other treatment. Cucurbit[8]uril was synthesized and purified following literature’s protocol.^37^

### Sample Preparation for Electrochemical Measurements

Sodium phosphate buffer (pH 7.0) was selected as supporting electrolyte in electrochemical measurements, which was prepared from sodium phosphate monobasic (NaH_2_PO_4_) and sodium phosphate dibasic heptahydrate (Na_2_HPO_4_·7H_2_O) in deionized water (deionized water, 18.2 MΩ·cm). Compared the electrochemical results obtained in different concentration of electrolyte, for example, 6.25 mM, 20 mM, 50 mM, and 100 mM, the concentration of electrolyte was chosen as 6.25 mM after compromising between the solution conductivity and stability of complexes (seeing Supporting Information (SI) Figure S1 for details). pH value was periodically checked by FiveEasy F20 pH meter during the preparation.

One mM MV^2+^ solution was prepared by adding methyl viologen dichloride hydrate (12.9 mg, 0.05 mmol) into 50 mL sodium phosphate buffer solution (pH 7.0), followed by sonication for 5 min. CB8 (66.5 mg, 0.05 mmol) was added to form 1 mM CB8-MV^2+^ complex. To facilitate the complexation, the solution was placed in sonication bath for more than 5 h before subsequent steps.

To form 1:1:1 ternary complex of CB8-MV^2+^-G2, 25 different second guests were added at a concentration of 1 mM into a 1 mM CB8-MV^2+^ solution. For the series of cyclohexene, cyclohexane, 1,3-cyclohexadiene, 1,4-cyclohexadiene, and benzene, which are of low water solubility, large excess of them were added. In particular, 1 mL of these guests was added into 10 mL of 1 mM CB8-MV^2+^ solution. All ternary complex sample solutions were then placed in a sonication bath for more than 4 h to ensure complete dissolution before electrochemical measurements.

### Electrochemical Measurements

Unless stated otherwise, all electrochemical experiments were carried out at room temperature with Gamry Interface 1010E workstation in a typical three-electrode electrochemical cell, which consists of (1) reference electrode: leakless Ag/AgCl electrode (*d* = 5 mm), (2) counter electrode: platinum plate electrode (6.5 mm × 6.5 mm), and (3) working electrode: gold disk electrode (*d* = 2 mm). To eliminate dissolved oxygen in solution, all samples were degassed by purging nitrogen gas for more than 10 min prior to the measurement.

The cyclic voltammetric (CV) measurements of all analytes were conducted by cycling potential from −200 mV to −800 mV at scan rates of 10 mV/s, 20 mV/s, 50 mV/s, and 100 mV/s for five cycles. The square wave voltammetric (SWV) measurements were performed from 0 mV to −800 mV with step size of 2 mV, pulse size of 25 mV and frequency of 5 Hz. The equilibrium time was set as 15 s for each measurement. All measurements were done with these parameters unless stated otherwise.

### ^1^H NMR

All ^1^H NMR measurements were performed on Bruker Avance III 400 MHz instrument and ^1^H NMR spectra were collected at room temperature. All ^1^H NMR samples were prepared at concentration of 1 mM with deuterium oxide (D_2_O) as solvent.

### Optimization of Molecular Models

Molecular models were optimized using MMFF94 in Chem3D followed by full optimization at wB97XD/6-31G* and CPCM/wB97XD/6-31G* level of theory using Gaussian 09. CPCM implicit water model was employed to approximate the solvent effects. Meanwhile the dispersion-corrected DFT functional wB97XD was chosen to accurately estimate the van der Waals interactions, which are expected to contribute greatly to the stability of the host-guest complexes. Energies of optimized structures were utilized to calculate energy change in redox processes and association–disassociation processes. All DFT optimization was performed on the UCL Myriad high performance computing facility (Myriad@UCL) and the UK Materials and Molecular Modeling Hub.

### Ab Initio Molecular Dynamics

Energy and entropy values were computed from 30 ps trajectories obtained for the range of ternary complexes. The software CP2K was used together with the PM6-D3 semiempirical DFT model at 1 fs time step under vacuum.^38,39^ Systems were equilibrated for 10 ps using a CSVR thermostat using a 100 fs time constant within the NVT ensemble and a target temperature of 300 K. CB8-MV^+•^-based ternary complexes were treated using unrestricted Kohn–Sham with a charge of 1 and a multiplicity of 2. CB8-MV^2+^-based ternary complexes were assigned a charge of 2 and a multiplicity of 1. After thermal equilibration, the thermostat was removed, and the system allowed to propagate in time for 30 ps in the NVE ensemble. The ASPC extrapolation scheme was used to improve energy conservation over the trajectory.^40^ The average energy for each system was obtained by averaging the potential energy readings for the system at each 10 time step. The configurational entropy was obtained using spectrally resolved estimation of entropy. All configurations from a trajectory were first RMSD aligned using Open Babel then the power spectrum of the Cartesian coordinates of the atoms was computed and truncated after 4000 cm^–1^. Eventually the power spectrum was used to integrate the estimate of the configurational entropy value.^41^ Translational and rotational entropy values included in the computation of the Gibbs free energy were obtained using Gaussian 16.

## Results and Discussion

The formation of CB8-MV^2+^-G2 1:1:1 heteroternary complex was performed by two sequential steps as shown in Figure 1a. In particular, CB8 formed a 1:1 binary complex with electron-deficient MV^2+^ via ion-dipole interactions between its electron-rich carbonyl portal and the positively charged MV^2+^. Then, 1 equiv of electron-rich G2 was introduced to form ternary complex with CB8-MV^2+^, where hydrophobic effects of G2 and charge transfer interactions serve as major driving forces for complexation. The complexation was evidenced by ^1^H NMR spectroscopy (Figure 1b), consistent to previous reports.^32^ Compared to free MV^2+^, chemical shifts of the pyridinium protons in CB8-MV^2+^ show an upfield shift, which is caused by the shielding effect of CB8. Moreover, they further shift to the higher field region and broaden after introducing 1 equiv of a sample G2, 2-naphthol (2NP), verifying the formation of a 1:1:1 CB8-MV^2+^-2NP ternary complex.

The corresponding electrochemical behaviors of free MV^2+^, 1:1 CB8-MV^2+^, and 1:1:1 CB8-MV^2+^-2NP were studied by cyclic voltammetry (Figure 1c). It is well-documented that dicationic MV^2+^ can undergo two consecutive reversible reduction processes, resulting in MV^+•^ and MV^0^, respectively.^34,35^ Here, we mainly focus on the first redox couple, that is, MV^2+^/MV^+•^, whose reduction potential was measured as −624.8 mV vs Ag/AgCl at 10 mV/s scan rate in CV mode. After adding 1 equiv of CB8 to form the CB8-MV^2+^ complex, the reduction potential shifts significantly to the positive direction to −494.8 mV, indicating that the reduction process of MV^2+^ becomes easier upon encapsulation by CB8. This observation is consistent with those reported by Kim et al., who showed that the positive shift is due to the reversible formation of stable 1:2 CB8-2MV^+•^ complexes after the one-electron reduction of MV^2+^ in the presence of equimolar CB8.^34^

Interestingly, in the presence of 2NP in the CB8-MV^2+^ system, the reduction potential of the resultant ternary complexes shifts negatively with respect to that of the CB8-MV^2+^. In particular the reduction potential shift displays an asymptotic growth as the amount of 2NP increases until 1 equivalence and remains stable in excess of 2NP (SI Figure S2). Hence, 1 equivalence of G2 was chosen in our following study, except otherwise specified. In the case of CB8-MV^2+^-2NP, the reduction potential shifts negatively to −577.9 mV (Figure 1c). This initial result suggests that the presence of a G2 and the subsequent formation of ternary complexes could hinder the reduction of the encapsulated MV^2+^, compared to the case of CB8-MV^2+^. Assuming the end products of reduction to be the stable CB8-2MV^+•^ complexes and free G2 molecules, the overall reaction is effectively an electrochemically induced dissociation of the CB8-MV^2+^-G2 ternary complexes. We therefore hypothesize that the magnitude of electrochemical hindrance (i.e., the shift in reduction potential with respect to that of CB8-MV^2+^, Δ*V*_G2_ in Figure 1c) should correlate to the thermodynamic stability (i.e., the binding constant *K*_G2_) of a particular ternary complex, and that MV^2+^ can serve as a redox probe for rapid estimation of the binding constant.

**Figure 2:**
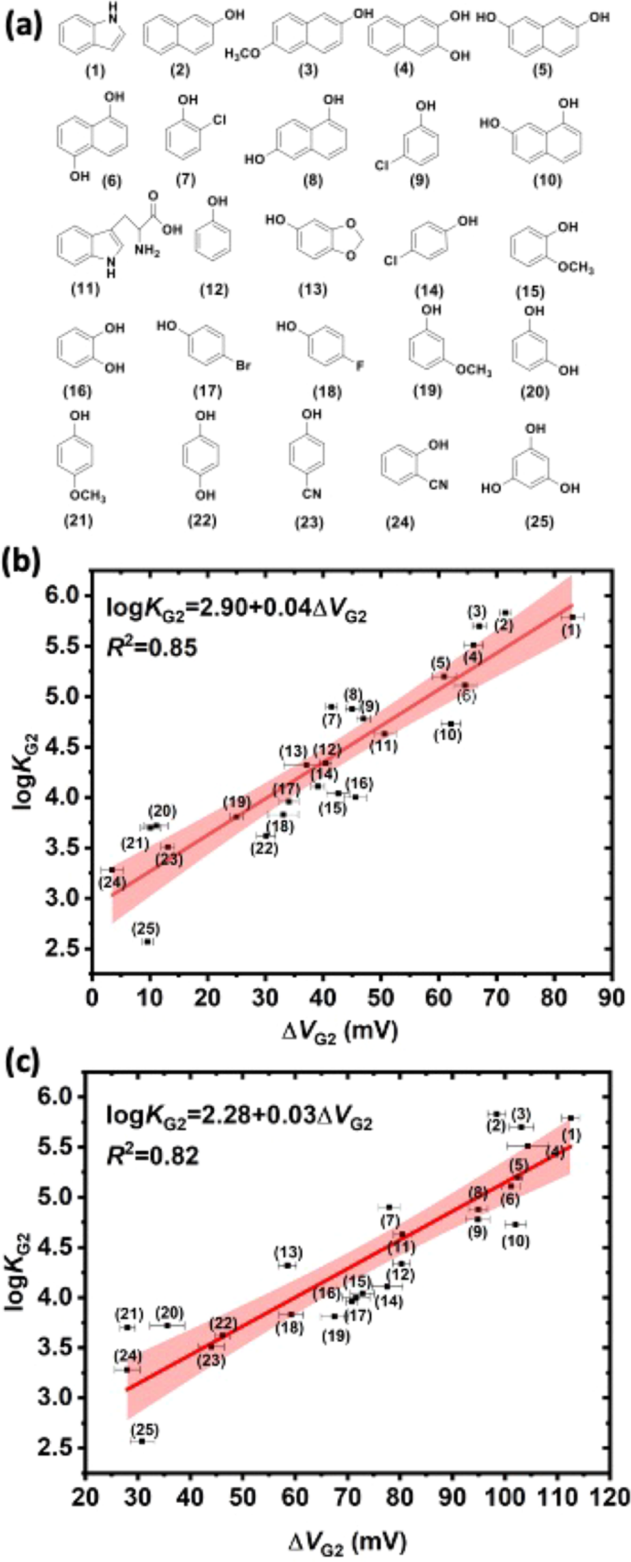
(a) Molecular structures of 25 reference G2 in descending order of reported ITC-determined *K*_G2_.^32^ Linear regression plots of log*K*_G2_ against the reduction potential shift Δ*V*_G2_ of CB8-MV^2+^-G2 ternary complexes measured in (b) cyclic voltammetric mode and (c) square wave voltammetric mode. Each data point is marked by a number that corresponds to the molecular structure in (a). Regression equations and *R*^2^ values are shown on the plots. Red line: linear regression plot. Pink band: 95% confidence band. Note that the regression equations are empirical in nature. The actual correlation should show boundary conditions of (i) when log*K*_G2_ → −infinity (i.e., *K*_G2_ = 0), Δ*V*_G2_ → 0 and (ii) when log*K*_G2_ → + infinity, Δ*V*_G2_ → constant. Hence, the correlation curve should not be linear throughout the entire range.

To validate the above hypothesis, we measured the electrochemical response by CV and SWV for a range of CB8-MV^2+^-G2 ternary complexes. Twenty-five electron-rich aromatic species were chosen as reference G2, whose chemical structures are shown in Figure 2a in descending order of the reported *K*_G2_ obtained by ITC.^32^ Electrochemical measurements were performed in sodium phosphate buffer solution (pH 7.0) (SI Figures S3 and S4), which is the same solvent condition as the corresponding ITC experiments to avoid introducing inconsistency between two sets of data.

ITC binding constants (log*K*_G2_) are plotted against Δ*V*_G2_ for the 25 reference G2 (Figure 2b for CV data and Figure 2c for SWV data). It is noted that the log*K*_G2_ values determined by ITC were assumed to be the true values with negligible errors in order to eliminate the methodological uncertainty of ITC in our electrochemical results (see SI Figure S5 for fitting with experimental error in log*K*_G2_ taken into account). Notably, we discovered a simple linear relationship between log*K*_G2_ and Δ*V*_G2_ with high *R*^2^ values of 0.85 (CV) and 0.82 (SWV), see SI Figure S6 for residual analysis of the fitting. *R*^2^ values show an increasing trend as the scan rate decreases, suggesting that at a low scan rate (e.g., 10 mV/s) the system approaches a steady state which can well represent the thermodynamic equilibrium of the corresponding host-guest complexation (SI Figure S7). This is consistent to the fact that CV peak potential values alone do not exhibit thermodynamic significance, unlike half-wave potentials, and thus a low scan rate is of crucial importance to the credibility of the data. Meanwhile the empirical linear regression equations of CV and SWV show almost the same slope values, indicating the consistency between the two measurement modes. The slightly smaller y-intercept in the SWV fitting is due to the generally larger Δ*V*_G2_ as obtained by this technique, which can be attributed to the minor contribution from the shifted anodic peak to the overall SWV signal (SI Figure S8).^42^

The linear correlation shown in Figure 2b,c enables rapid estimation of log*K*_G2_ of unknown ternary complexes based on the measured Δ*V*_G2_ from CV or SWV which can be obtained in less than 10 min (e.g., in CV mode, scan rate = 10 mV/s, range: from −200 mV to −800 mV, number of scan cycles = 5), excluding sample preparation time. In contrast to titration techniques, our electrochemical approach requires only a single measurement on a sample solution of ternary complexes at 1:1:1 ratio. The precision of our measurement can be assessed by considering the standard deviation of the measured Δ*V*_G2_ which has an averaged value of 1.66 mV for CV and 1.06 mV for SWV over the 25 G2 (five measurements each), corresponding to precision in log*K*_G2_ of 0.06 for CV and 0.03 for SWV. Remarkably, the precision of our approach rivals that of ITC measurements. Assuming the log*K*_G2_ determined by ITC as the true value, the accuracy of log*K*_G2_ determined by our electrochemical scheme can be estimated as the root mean squared deviation of ITC-determined log*K*_G2_ from the regression equations, and is given as ±0.32 and ±0.35 based on the CV and the SWV data, respectively. This accuracy range is considered adequate for high-throughput screening and ranking of binding constants of unknown second guests.

When studying unknown G2 candidates, it is not uncommon to encounter bulky molecules that are borderline too big to form ternary complexes with CB8 and MV^2+^. This scenario will typically result in the formation of CB8-G2 binary complexes and displacement of MV^2+^ from the CB8 cavity. In some titration methods (e.g., UV–vis titration), such host-guest exchange could be incorrectly identified as a binding event with a false-positive binding constant. Here, we tested our electrochemical scheme against this scenario using 1-adamantylamine (AdNH_2_) as a model bulky guest. Upon increasing equivalence of AdNH_2_, a convoluted reduction peak of CB8-MV^2+^ and free MV^2+^ can be observed in the SWV data, indicating the occurrence of host-guest displacement rather than a binding event (SI Figure S9). The shape of the convoluted peak can be easily recognized by human eyes or a computer algorithm, which can therefore reduce the likelihood of generating false-positive binding results.

To investigate the electrochemical mechanism and the origin of the observed correlation between Δ*V*_G2_ and log*K*_G2_, we have performed a mechanistic analysis on the electrochemical data. First of all, we hypothesize that in the CB8-MV^2+^-G2 system, the redox probe MV^2+^ exists predominantly in the encapsulated form, and electron transfer occurs directly from the electrode surface to the ternary complex. The former assumption is supported by the exceptionally high binding constants of CB8-based ternary complexes (log*K* = 8–11).^17^ Indeed, electrochemical measurements, especially SWV, offer high sensitivity to detect the existence of free MV^2+^ (SI Figure S10). Nevertheless, we did not observe any signal from free MV^2+^ under our experimental conditions at 1:1:1 host-guest ratio. The latter hypothesis receives support from the fact that MV^2+^ exhibits fast heterogeneous electron-transfer kinetics, and that efficient electron transfer of CB7-encapsulated MV^2+^ has been reported in the literature.^43^ We therefore propose that the observed reduction peaks of MV^2+^ in CV and SWV can be attributed to an *E*_r_*C*_r_ mechanism,^42^ where reversible electron transfer (eq 1) is followed by a subsequent step of reversible homogeneous chemical change, that is, host-guest exchange in our case (eq 2).
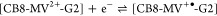
1
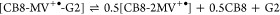
2

Closer inspection on the cyclic voltammograms at a scan rate of 10 mV/s reveals that the peak current ratio (i.e., *i*_a,p_/*i*_c,p_, where *i*_a,p_ and *i*_c,p_ are the anodic and cathodic peak current, respectively) becomes slightly larger for CB8-MV^2+^ (1.32) compared to free MV^2+^ (1.07), which can be attributed to the binding of CB8, and therefore enrichment and retainment of MV^2+^/MV^+•^, on the Au electrode surface.^14,44^ It is noted that the peak current ratio has a theoretical maximum value of 1. The observed larger-than-1 values come from the overestimation of the cathodic current at switching potential *i*_s,p_ in the employed Nicholson method,^45^ due to the onset of the hydrogen evolution reaction occurring at the switching potential (see SI Figure S11 for details).

Meanwhile we observed an increase in the peak-to-peak potential splitting (Δ*E*_p_) for CB8-MV^2+^ (125 mV) and all CB8-MV^2+^-G2 complexes (92 to 217 mV) compared to free MV^2+^ (83 mV), indicating that the anodic reaction in the reverse scan becomes more kinetically hindered upon CB8-complexation. This observation aligns to the proposed formation of the kinetically stable 1:2 CB8-2MV^+•^ complex via host-guest exchange (eq 2) where direct oxidation is unfavorable due to the transient formation of a highly (3+) charged complex.

Additional insights can be extracted by analyzing the scan-rate dependence of the peak current ratio in the cyclic voltammograms (SI Figure S11). Interestingly, we observe a general increase in peak current ratio upon increase in scan rate (from 10–100 mV/s), which suggests an irreversible homogeneous chemical change (i.e., *E*_r_*C*_i_ mechanism). This paradoxological finding highlights the exceptional kinetic stability of CB8-2MV^+•^ complex which results in a very slow backward reaction rate in eq 2 relative to the measurement time scale during the reverse scan, making the host-guest exchange seemingly irreversible. This scenario is also consistent with the observed increase in peak-to-peak potential splitting upon an increasing scan rate (SI Figure S12). Furthermore, we also note significant scan-rate dependence in the shape of CV especially for ternary complexes with a large *K*_G2_. In particular, a bulge on the negative side of the main anodic peak appears at higher scan rates for complexes of strongly binding G2, for example, indole and 2NP (SI Figure S13). This minor anodic peak can be attributed to the direct oxidation of the less stable CB8-MV^+•^-G2,^46^ indicating that a strongly binding G2 can indeed compete with the formation of the more stable CB8-2MV^+•^ complex, and the host-guest exchange of strongly binding G2 follows slow kinetics compared to that of weakly binding G2 which exhibits a highly symmetric CV without the bulge across various scan rates. We note that the detailed electrochemical mechanism can be more complicated due to interplay between multiple redox active species under dynamic interconversion, and is beyond the scope of the current study.

Computational modeling based on density functional theory (DFT) was performed with an aim to obtain quantitative understanding into the correlation between Δ*V*_G2_ and log*K*_G2_. Molecular models were optimized using MMFF94 followed by full optimization at the CPCM/wB97XD/6-31G* level of theory (see Supporting Information for computational method details). CPCM implicit water model was employed to approximate the solvent effects. Meanwhile the dispersion-corrected DFT functional wB97XD was chosen to accurately estimate the van der Waals interactions, which are expected to contribute greatly to the stability of the host-guest complexes. In particular, we first attempted to calculate the energy change of the reduction process (eq 1 and SI eq S2) based on the optimized structures. Unexpectedly, the computed energy changes of the 25 reference ternary complexes do not show any correlation to their ITC-determined log*K*_G2_ (see SI Figure S14).

As it is expected that CBs’ cavities possess an intricate energy landscape that cannot be fully accounted for using energies and structures of a local minimum,^47^ a further attempt using ab initio molecular dynamics at the PM6-D3 level of theory was made but did not reveal any satisfying correlation either (SI Figure S15). In particular, based on 30 ps trajectories of the reference ternary complexes in their oxidized and reduced states in vacuum, the energy change in the reduction step was computed from the time-averaged energy of a given complex, while the configurational entropy was estimated using spectrally resolved estimation.^41^ It is revealed that the magnitude of the change in configurational entropy upon reduction can be greater than 10 kcal/mol at *T* = 300 K which is considered non-negligible. However, no systematic trend in configurational entropy upon reduction can be observed. Similarly, no significant trend could be extracted from the energy difference upon reduction of the ternary complexes, while their spread is larger than that in the case of the wB97XD optimization. As shown in SI Figure S15, no high quality correlation could be obtained from the values of the free energy of reduction versus experimental log*K*_G2_, rendering improbable a direct modulation of the standard redox potential of CB8-MV^2+^ by the inclusion of G2.

These computational results indicate that the Δ*V*_G2_-log*K*_G2_ correlation should be rooted in the subsequent electrochemically induced supramolecular dissociation and host-guest exchange events (eq 2). Indeed, the previously reported shift in redox potential in the CB8-MV^2+^ complex is also attributed to the redox induced host-guest rearrangement, rather than the relative stability of the redox couple itself. Hence the observed Δ*V*_G2_ reflects the combined free energy difference of eq 1 and 2, consistent with the proposed coupled *E*_r_*C*_r_ mechanism. Gratifying to observe, the computed energy change of eq 2 shows excellent correlation (*R*^2^ = 0.95) to that of the computed binding energy between CB8-MV^2+^ and G2 at the CPCM/wB97XD/6-31G* level of theory (SI Figure S16b). This finding implies that a strong G2 for CB8-MV^2+^ is also a strong G2 for CB8-MV^+•^. Hence the electrochemical reduction of CB8-MV^2+^-G2 will not perturb the relative binding affinity within a series of G2, but will merely produce a competitive binding pathway with CB8-2MV^+•^ as the stable product.

In the context of the Nernst Equation (*E* = *E*_0_ – (*RT*/*F*) ln ([Red]/[Ox])), the observed reduction potential *E* depends on the standard reduction potential *E*_0_, which is a function of the overall free energy change, and the ratio of the concentrations of the reduced and oxidized forms [Red]/[Ox] of a given redox couple. In the case of CB8-MV^2+^, it has previously been shown that a 1:2 CB8-2MV^+•^ complex could be formed that dramatically eased the reduction process of MV^2+^.^34^ This observation can be attributed to the efficient sequestration of the active reduced species CB8-MV^+•^ via host-guest exchange to form the less active CB8-2MV^+•^ together with empty CB8. Indeed, depletion in the active reduced species would increase the observed reduction potential. Here we argue that the binding strength of G2 with CB8-MV^2+^ modulates the ease with which the 1:2 complex can be formed. By hindering the formation process of the 1:2 complex, a strong G2 could therefore push the reduction potential of the encapsulated MV^2+^ closer to its value in solution in the absence of CB8.

As a proof-of-concept application of our electrochemical approach, we investigated the binding affinity of a series of cyclic hydrocarbons, namely (i) cyclohexane, (ii) cyclohexene, (iii) 1,3-cyclohexadiene, (iv) 1,4-cyclohexadiene, and (v) benzene. All these G2 candidates are known to be volatile and some of them exhibit low solubility in water, so it is impractical to prepare their aqueous samples with accurate concentrations, making titration measurements inviable. In our case, 1:1:1 ternary complexes (CB8-MV^2+^-G2) were prepared by mixing excess G2 with an aqueous solution of CB8-MV^2+^. As illustrated in the case of 2NP, the value of Δ*V*_G2_ becomes stable when the equivalence of G2 is greater than 1 (SI Figure S2).

**Figure 3:**
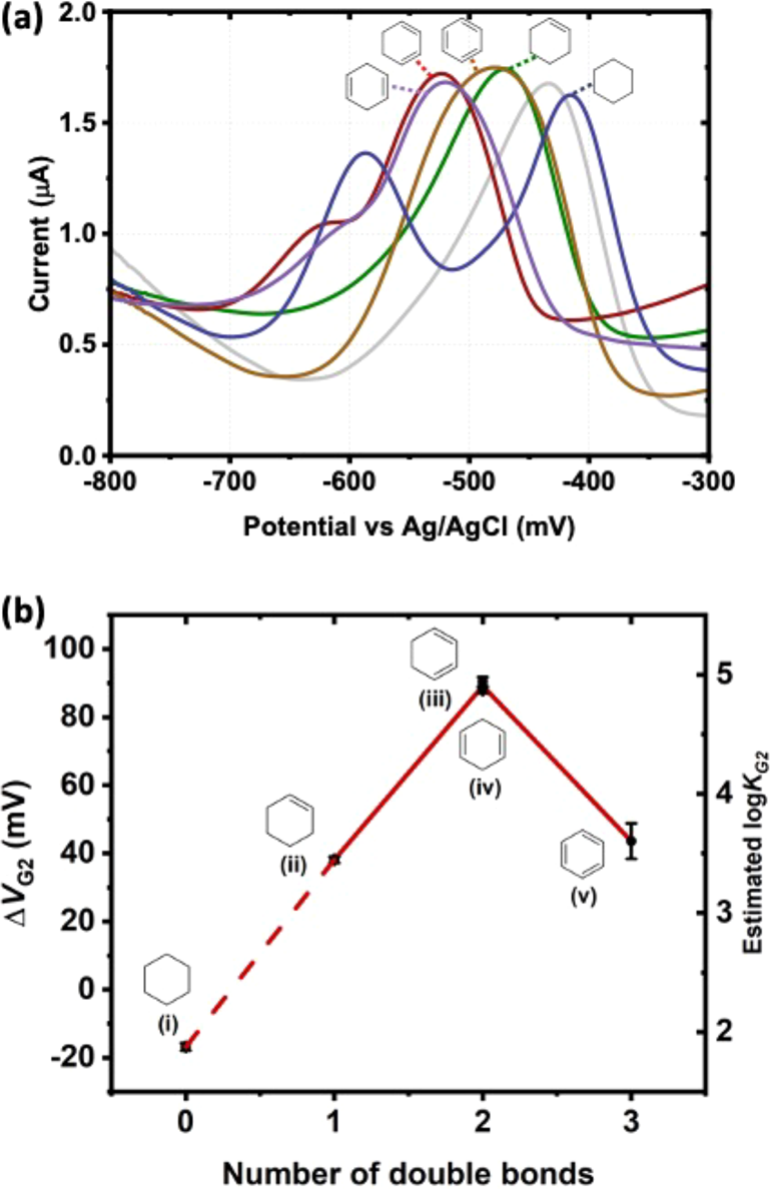
(a) Square wave voltammetric response of CB8-MV^2+^-G2 ternary complexes and CB8-MV^2+^ (gray). G2 = (i) cyclohexane (blue), (ii) cyclohexene (green), (iii) 1,3-cyclohexadiene (red), (iv) 1,4-cyclohexadiene (purple) and (v) benzene (brown); (b) Plot of Δ*V*_G2_ and the estimated log*K*_G2_ of CB8-MV^2+^-G2 complexes against number of double bonds in G2.

Indeed, we observed a shift in reduction peaks caused by the presence of G2 in both SWV and CV data (Figure 3a, SI Figure S17b). The measured Δ*V*_G2_, together with the estimated log*K*_G2_, were then plotted against the number of double bonds in G2 (Figure 3b, SI Figure S17c). Except for cyclohexane, other four six-membered cyclic hydrocarbons exhibit log*K*_G2_ values between 3.5 and 5.0 which are consistent with the values of other G2 with similar structures.^17^ Meanwhile the estimated log*K*_G2_ shows a general trend of increase with the number of double bonds in G2, which can be attributed to the strengthening of van der Waals and charge transfer interactions between π-electrons on MV^2+^ and G2. The nonrotatable double bonds also make the G2 less flexible and thus reduce the entropic penalty of complexation. The drop in estimated log*K*_G2_ for benzene should be due to its high solubility in water that disfavors the host-guest binding, consistent with the previous study about the importance of solvation energy in the formation of CB8 ternary complexes.^32^ Interestingly, the SWV and CV in the case of cyclohexane show two reduction peaks at −586.8 mV (SWV) and −415.6 mV (SWV). By comparing to the SWV of free MV^2+^ and CB8-MV^2+^ as shown in SI Figure S18, the peak at −586.8 mV can be assigned to free MV^2+^ while the peak at −415.6 mV can correspond to either CB8-MV^2+^ (SWV peak at −466.7 mV) or CB8-MV^2+^-cyclohexane with an estimated log*K*_G2_ of ∼1.8 if it forms at all. The presence of free MV^2+^ suggests the possibilities of the formation of CB8-cyclohexane complexes or precipitation of CB8. Thus, despite the suitable molecular size and hydrophobicity of cyclohexane, our results indicate that there is very weak or no binding between cyclohexane and CB8-MV^2+^, which is consistent with its lack of π-electrons and its molecular flexibility. The formation (or the lack of it) of the ternary complex has been verified by ^1^H NMR (SI Figure S17a).

## Conclusions

We report a simple and effective electrochemical scheme for estimating the binding constant of CB8-MV^2+^-G2 heteroternary complexes, achieving high precision (±0.03) and practical accuracy (±0.32) in log*K*_G2_ as well as short measurement time of less than 10 min which is significantly faster than conventional titration methods (e.g., ∼5 h for ITC). This approach is underpinned by the discovery of a linear correlation (*R*^2^ = 0.85) between log*K*_G2_ and Δ*V*_G2_. Mechanistic investigations using experimental and computational techniques reveal that this correlation stems from the dynamic host-guest exchange events occurring after the electron transfer step. Finally, we illustrated the versatility and robustness of our approach by investigating a series of six-membered cyclic hydrocarbons as G2 candidates and revealing the trend of log*K*_G2_, which cannot be directly determined otherwise due to their low aqueous solubility and high volatility. The feature-rich electrochemical data also allows us to extract multiple states of the redox probe (MV^2+^) in the system and aid minimizing false-positive binding results. We expect that this approach can be readily extended to studying other redox-active host-guest systems such as those involving MV^2+^-based cyclophane hosts, and opens up new possibilities in designing high-throughput schemes for binding constant determination.

## Supporting Information Available

CV and SWV of MV^2+^, CB8-MV^2+^ and CB8-MV^2+^-2NP; Titration of CB8-MV^2+^-2NP; CV and SWV of CB8-MV^2+^-G2; Linear regression of log*K*_G2_ against Δ*V*_G2_; Residual analysis of linear regression; ^1^H NMR and SWV of CB8-MV^2+^-AdNH_2_; Peak current ratio and peak-to-peak splitting of CB8-MV^2+^-G2, CB8-MV^2+^ and MV^2+^ in CV; Computed energy change and binding energy plots; ^1^H NMR, CV and correlation plot of CB8-MV^2+^-hydrocarbons (PDF)
