# Supplementary material for: Rapid Estimation of Binding Constants for Cucurbit[8]uril Ternary Complexes Using Electrochemistry
Source: Anal Chem. 2021 Feb 17;93(9):4223–30. doi: 10.1021/acs.analchem.0c04887 (PMC8023530; doi:10.1021/acs.analchem.0c04887)
Supplement: Supplementary file 1 — ac0c04887_si_001.pdf [file ac0c04887_si_001.pdf]

Supporting Information

Rapid Estimation of Binding Constants for Cucurbit[8]uril Ternary Complexes Using Electrochemistry

Jia Liu,[a,b] Hugues Lambert,[a,b,c] Yong-Wei Zhang,[c] and Tung-Chun Lee*[a,b]

[a] Institute for Materials Discovery, University College London (UCL), Bloomsbury, London, WC1E 7JE, United Kingdom. Email: tungchun.lee@ucl.ac.uk

[b] Department of Chemistry, University College London (UCL), 20 Gordon Street, London, WC1H 0AJ, United Kingdom.

[c] Institute of High Performance Computing, 1 Fusionopolis Way, 138632, Singapore.

Table of Contents

1. SUPPORTING DATA AND FIGURES S3

Figure S1. Cyclic voltammetric response and square wave voltammetric response of MV^{2+}, CB8-MV^{2+} and CB8-MV^{2+}-2 naphthol at different concentration of electrolyte S3

Figure S2. Reduction potential shift (ΔV_{G2}) of 1 mM CB8-MV^{2+} in presence of various ratios of 2NP measured by square wave voltammetry........ S4

Figure S3. Cyclic voltammetric response of 25 different CB8-MV^{2+}-G2 ternary complexes........ S5

Figure S4. Square wave voltammetric response of 25 different CB8-MV^{2+}-G2 ternary complexes....... S7

Figure S5. Linear regression plots of $\log K_{G2}$ with experimental error of ITC against the reduction potential shift ΔV_{G2} of CB8-MV^{2+}-G2 ternary complexes measured in (a) cyclic voltammetric mode and (b) square wave voltammetric mode S7

Figure S6. Residual analysis of linear regression plots of $\log K_{G2}$ against reduction potential shift (ΔV_{G2}) of CB8-MV^{2+}-G2 ternary complexes S8

Figure S7. Linear regression plots of ITC-determined $\log K_{G2}$ against the reduction potential shift ΔV_{G2} of CB8-MV^{2+}-G2 ternary complexes measured in cyclic voltammetric mode at different scan rates..... S9

Figure S8. Overall square wave voltammetric response, corresponding cathodic curve and anodic curve of free MV^{2+}, CB8-MV^{2+} and CB8-MV^{2+}-2NP complex. S9

Figure S9. ^{1}H NMR spectra and square wave voltammograms of the CB8-MV^{2+} complex in the presence of various equivalents (0, 0.5, 1 and 2) of 1-adamantylamine ($AdNH_2$) and free MV^{2+} S10

Figure S10. Electrochemical behaviors of an aqueous solution containing 1.0 mM CB8 and 2.0 mM MV^{2+}........ S11

Figure S11. Peak current ratio of 25 different CB8-MV^{2+}-G2 ternary complexes, free MV^{2+} and CB8-MV^{2+} at different scan rates........ S12

Figure S12. Peak-to-peak separation of 25 different CB8-MV^{2+}-G2 ternary complexes, free MV^{2+} and CB8-MV^{2+} at different scan rates...S14

Figure S13. Cyclic voltammetric response of CB8-MV^{2+}-indole and CB8-MV^{2+}-2NP ternary complexes at different scan rates..S14

Figure S14. Scatter plots of computed energy change in the redox step against computed binding energy based on wB97XD/6-31G* and CPCM/wB97XD/6-31G*, and scatter plots of computed binding energy against the corresponding ITC-determined logK_{G2}..S15

Figure S15. Scatter plots of the computed enthalpy, entropy and Gibbs free energy change in the redox step based on molecular dynamics at PM6D3 level of theory against the corresponding ITC-determined logK_{G2}...S16

Figure S16. Correlation of the computed binding energy between CB8-MV^{2+} and G2 and the computed energy change of the post-reduction host-guest exchange...S16

Figure S17. ^{1}H NMR spectra and cyclic voltammetric response of CB8-MV^{2+} and CB8-MV^{2+}-G2 ternary complexes. G2 = cyclic hydrocarbons.Plot of cyclic voltammetric reduction potential shift (ΔV_{G2}) of CB8-MV^{2+}-G2 and predicted binding constants (logK_{G2}) of CB8-MV^{2+}-G2 complexes against number of double bonds in G2...S17

Figure S18. Square wave voltammograms of samples containing 1 mM free MV^{2+}, 1 mM CB8-MV^{2+}; 1 mM 1:1:1 of CB8, MV^{2+} and cyclohexane. S17

2. REFERENCES S18

1. Supporting data and figures

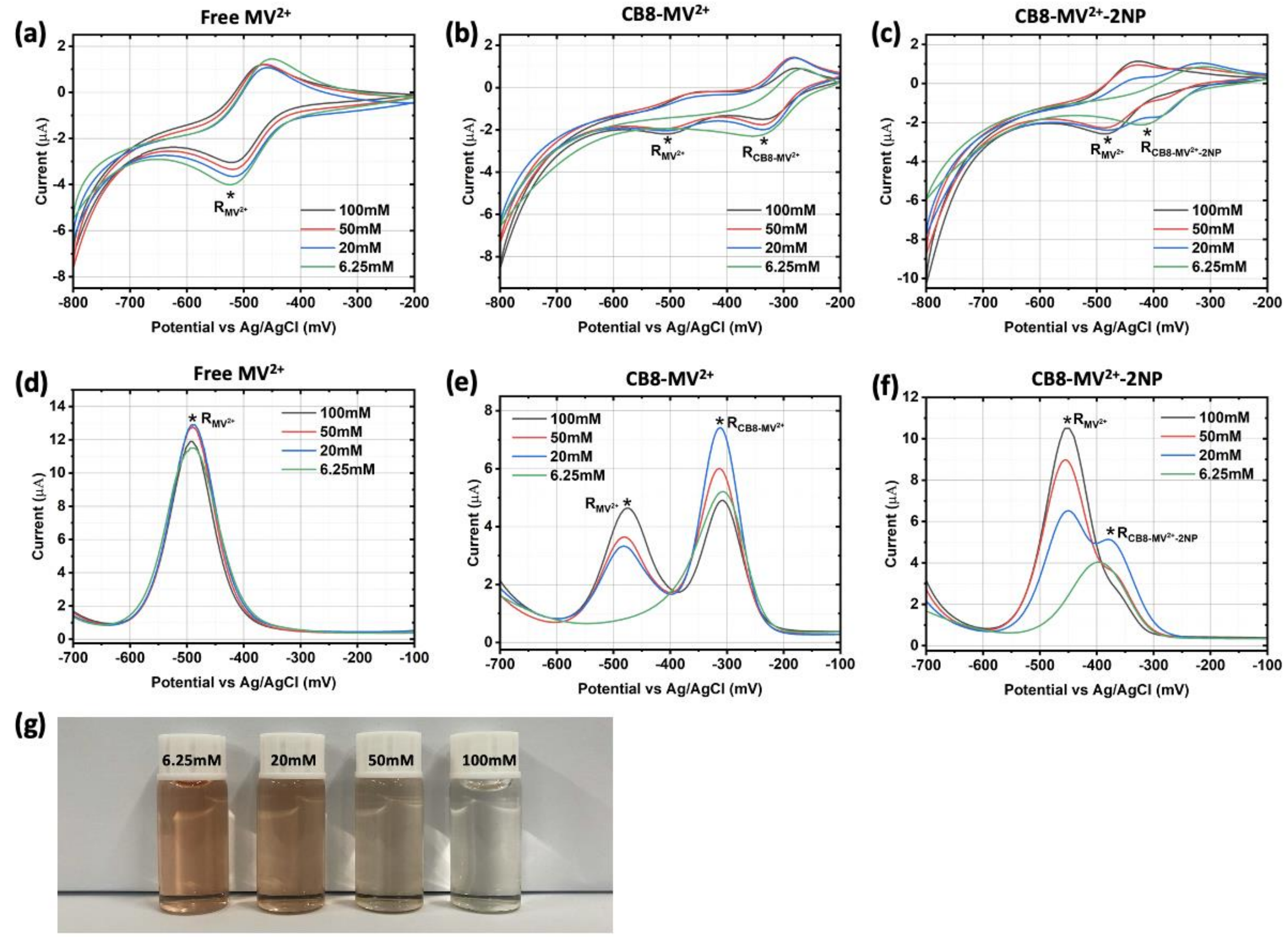

Figure S1. Cyclic voltammetric response and square wave voltammetric response of **(a, d)** MV^{2+}, **(b, e)** CB8-MV^{2+} and **(c, f)** CB8-MV^{2+}-2 naphthol (CB8-MV^{2+}-2NP) measured in different concentration (6.25 mM, 20 mM, 50 mM and 100 mM) of phosphate buffer solution (pH = 7.0) as electrolyte. **(g)** Photo of CB8-MV^{2+}-2NP samples prepared in different concentration of electrolyte as labelled. Scan rate of CV: 10 mV/s. Frequency of SWV: 5 Hz. Step size of SWV: 2 mV. Pulse size of SWV: 25 mV. Working electrode: gold disk (0.0314 cm^2). Reference electrode: Ag/AgCl. Electrochemical working station: CHI760E.

For fast redox couple MV^{2+}/$MV^{+\bullet}$, the peak-to-peak splitting in CV response decreases from 74 mV in 6.25 mM of electrolyte to 55 mV in 100 mM of electrolyte, while for CB8-MV^{2+} and CB8-MV^{2+}-2NP complexes, the stability of CB8-MV^{2+} and CB8-MV^{2+}-2NP complexes decreases significantly as the electrolyte concentration increases since Na^+ in solution is competitive to bind to CB8. As shown in CV and SWV results of CB8-MV^{2+} **(b, e)** and CB8-MV^{2+}-2NP **(c, f)**, another peak corresponding to free MV^{2+} appears when the concentration of electrolyte reaches 20 mM, and the ratio of I_p (CB8-MV^{2+})/ I_p (MV^{2+}) decreases as the concentration of electrolyte increases, indicating that concentrated electrolyte reduces the stability of CB8-MV^{2+} and CB8-MV^{2+}-2NP complexes and free MV^{2+} is generated by dissociation processes of complexes. In the case of CB8-MV^{2+}-2NP, when the electrolyte concentration reaches 100 mM, the peak corresponding to CB8-MV^{2+}-2NP disappeared completely and only the peak corresponding to free MV^{2+} was observed, suggesting that the stability of CB8-MV^{2+}-2NP decrease more

significantly than CB8-MV^{2+}. Meanwhile, the colour of CB8-MV^{2+}-2NP solution changes from flesh pink in 6.25 mM of electrolyte to almost colourless in 100 mM of electrolyte (**g**), implying the amount of ternary complexes decreases as the electrolyte concentration increases. Thus, taking both solution conductivity and stability of complexes into account, 6.25 mM is chosen as the concentration of electrolyte for all other electrochemical measurements of CB8-MV^{2+}-G2 ternary complexes.

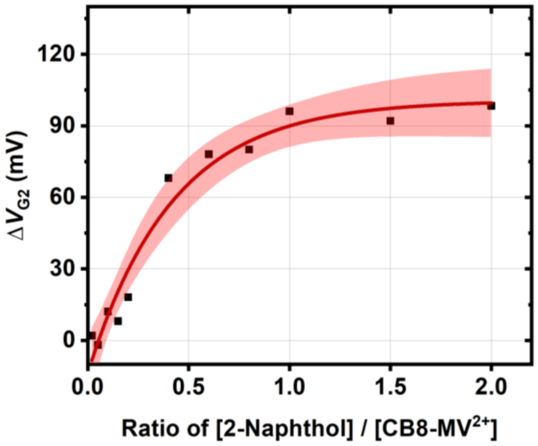

Figure S2. Reduction potential shift (ΔV_{G2}) of 1 mM CB8-MV^{2+} in presence of various ratios of 2NP measured by square wave voltammetry. Frequency: 5 Hz. Pulse Size E: 25 mV. Working electrode: gold disk (0.0314 cm^2). Reference electrode: Ag/AgCl. Medium: 6.25 mM phosphate buffer solution (pH = 7.0).

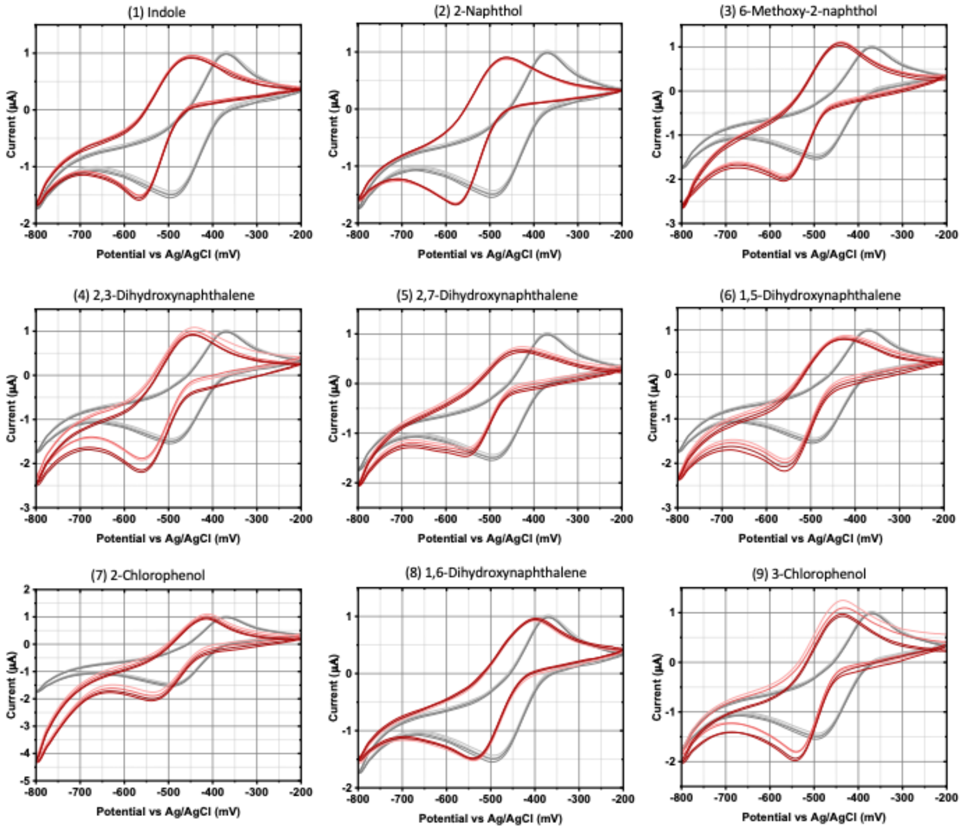

(Figure continues on page S5)

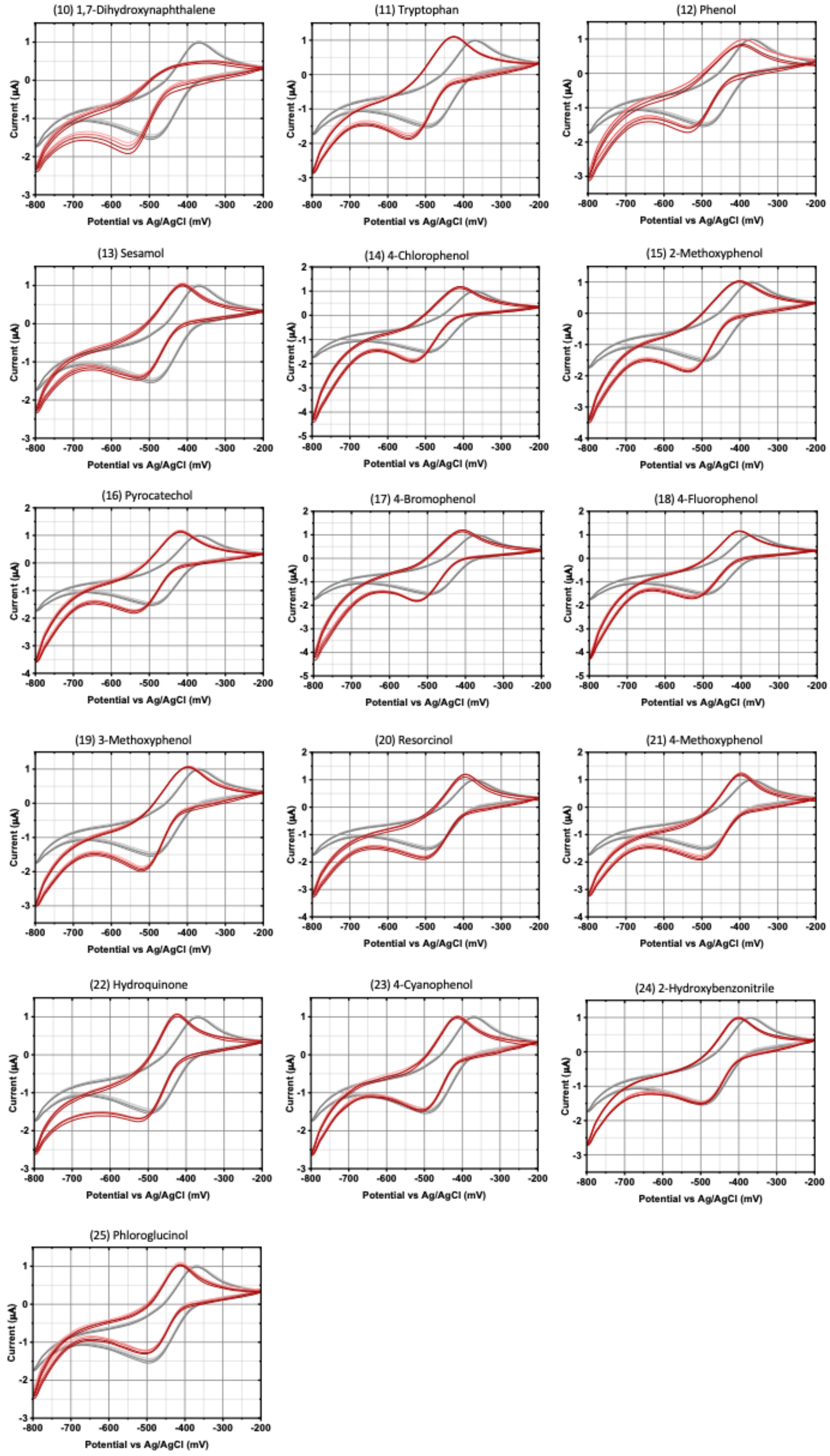

Figure S3. Cyclic voltammetric response of 25 different CB8-MV^{2+}-G2 ternary complexes (red lines) overlaid on CB8-MV^{2+} (grey lines) as reference. G2 is labelled on the top of each plot. Scan rate: 10mV/s. Scanning cycles: 5 cycles. Working electrode: gold disk (0.0314 cm^2). Reference electrode: Ag/AgCl. Medium: 6.25 mM phosphate buffer solution (pH = 7.0).

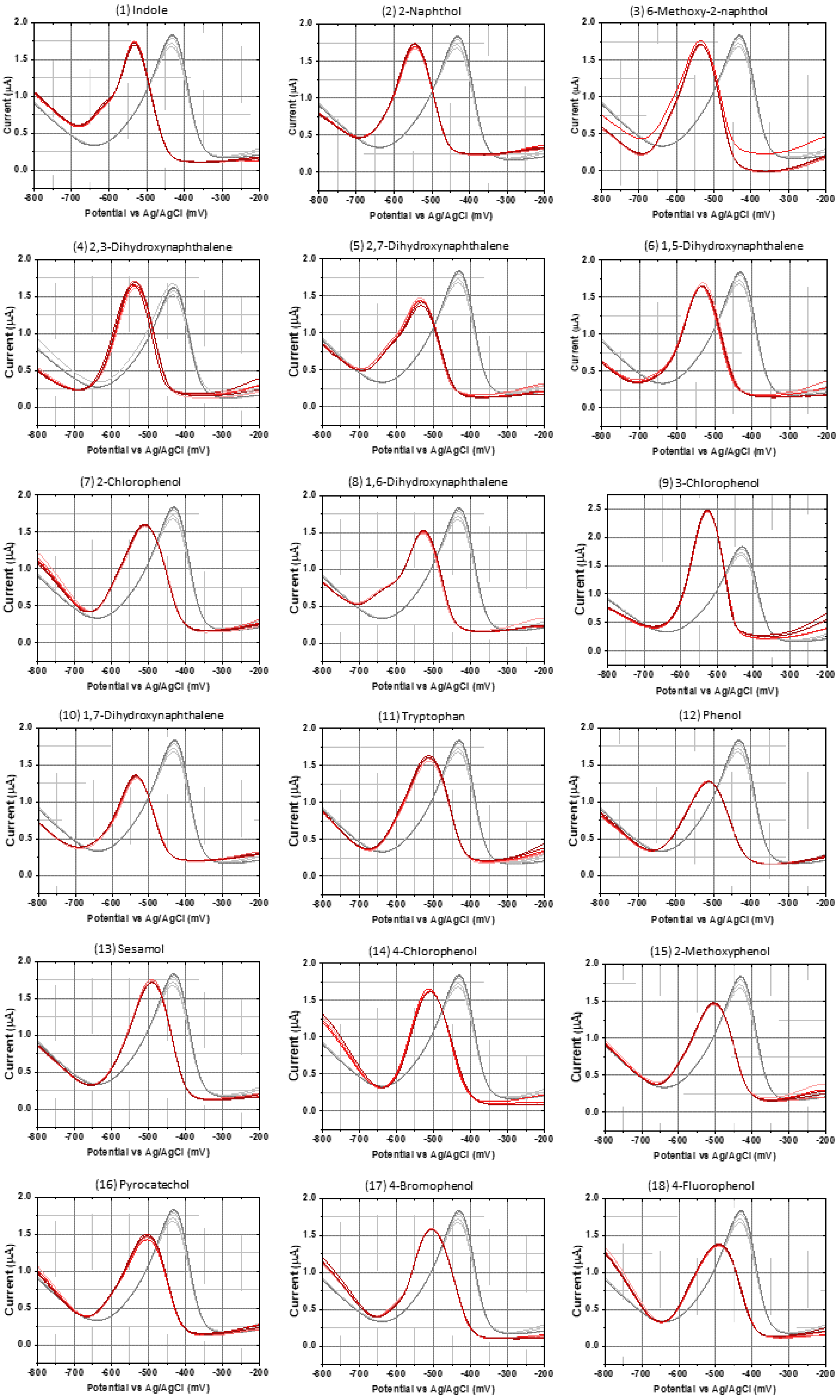

(Figure continues on page S7)

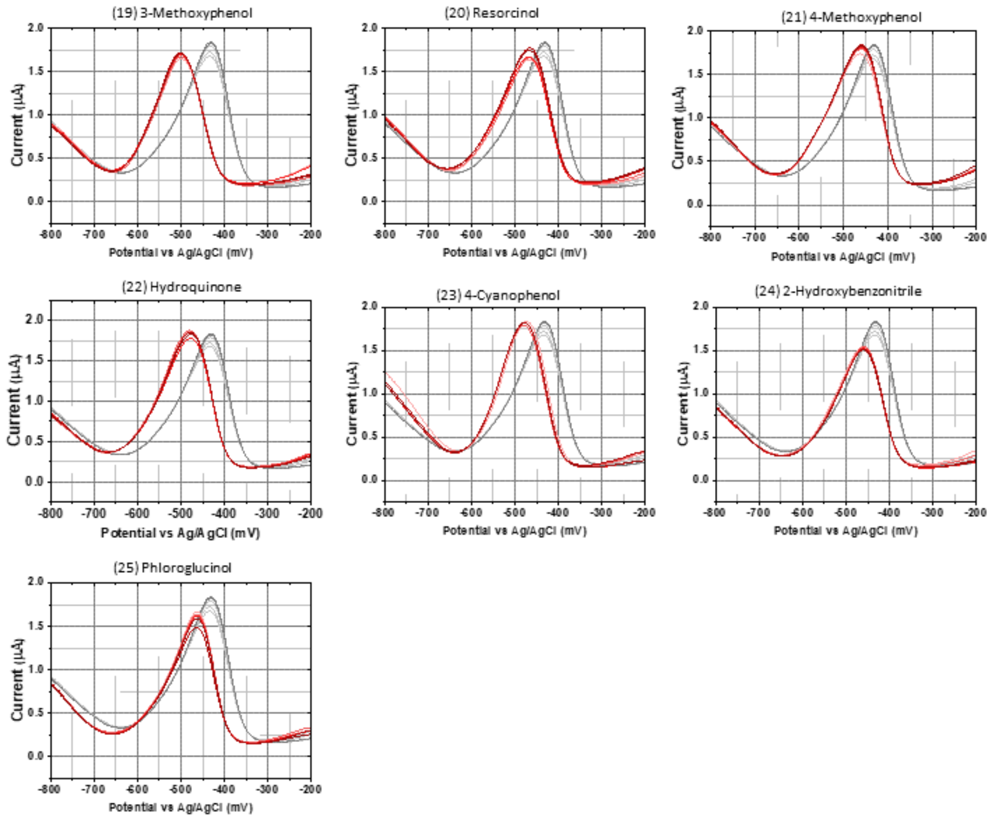

Figure S4. Square wave voltammetric response of 25 different CB8-MV^{2+}-G2 ternary complexes (red lines) overlaid on CB8-MV^{2+} (grey lines) as reference. G2 is labelled on the top of each plot. Step size: 2mV. Frequency: 5 Hz. Pulse Size E: 25 mV. Working electrode: gold disk (0.0314 cm^2). Reference electrode: Ag/AgCl. Medium: 6.25 mM phosphate buffer solution (pH = 7.0).

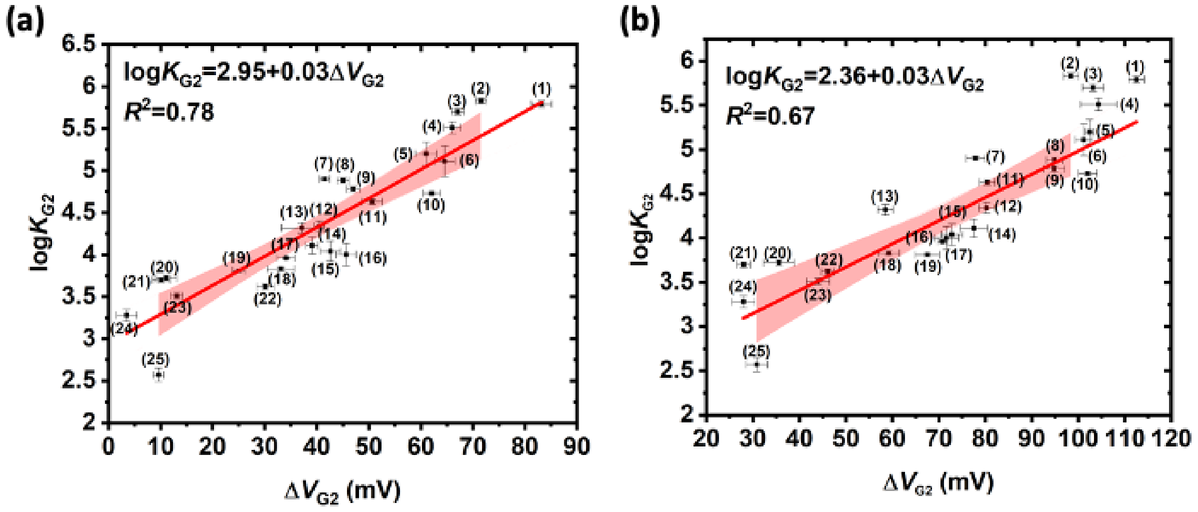

Figure S5. Linear regression plots of logK_{G2} with experimental error of ITC [S2] against the reduction potential shift ΔV_{G2} of CB8-MV^{2+}-G2 ternary complexes measured in **(a)** cyclic voltammetric mode and **(b)** square wave voltammetric mode. Each data point is marked by a number that corresponds to the molecular structure in Figure 2a of main text. Regression equations and R^2 values are shown on the plots. Red line: linear regression plot. Pink band: 95% confidence band.

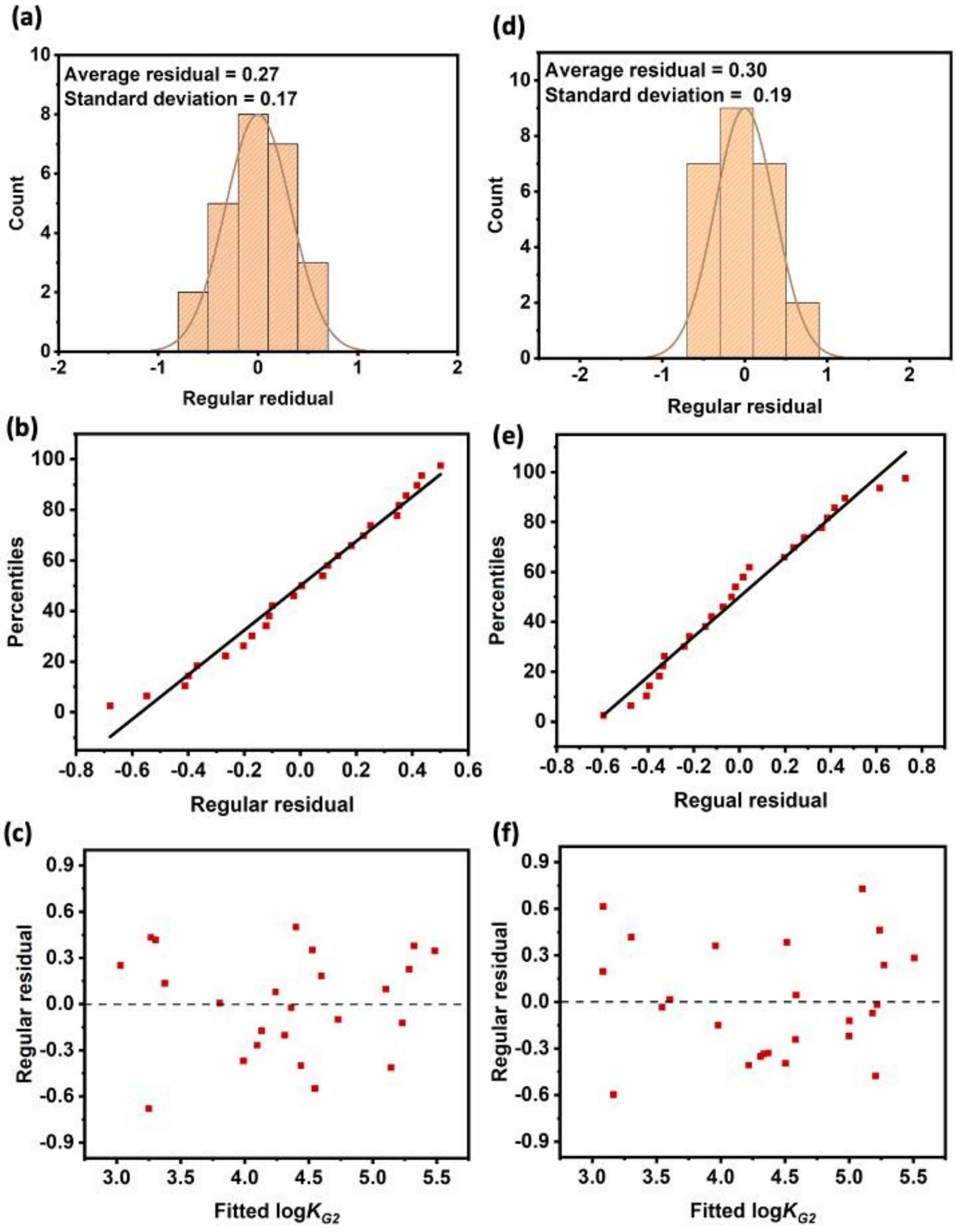

Figure S6. Residual analysis of linear regression plots of logK_{G2} against reduction potential shift (ΔV_{G2}) of CB8-MV^{2+}-G2 ternary complexes measured in **(a–c)** cyclic voltammetric mode and **(d–f)** square wave voltammetric mode. The corresponding regression plots are shown in Fig. 2b and c in the main text. Residual analysis supports the suitability of the regression model in addition to the R^2 value (0.85 for CV and 0.82 for SWV). In particular, the histograms (a) and (d) show a normal distribution of regular residual centered around zero with a narrow peak width. This is consistent to the linear trend in the percentiles against regular residual plots in (b) and (e). For the plots of regular residual against fitted logK_{G2} in (c) and (f), no trending bias in variance is observed across the entire range of logK_{G2}.

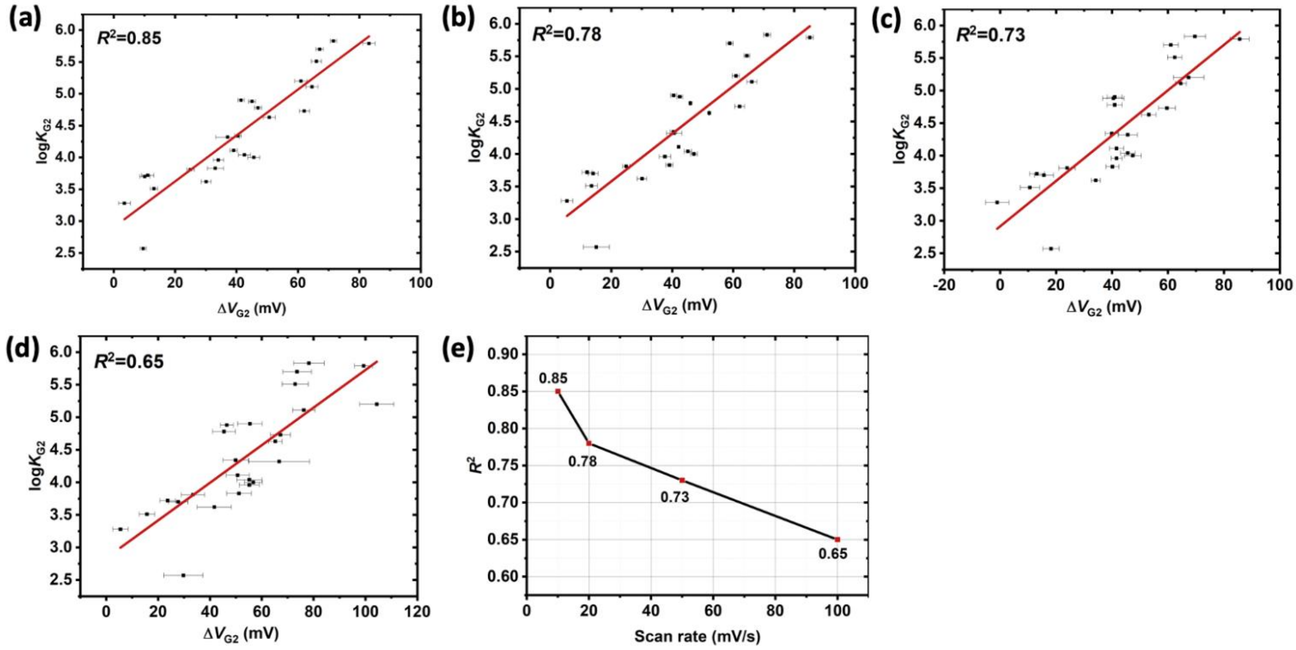

Figure S7. Linear regression plots of ITC-determined logK_{G2} against the reduction potential shift ΔV_{G2} of CB8-MV^{2+}-G2 ternary complexes measured in cyclic voltammetric mode at different scan rates of **(a)** 10mV/s; **(b)** 20mV/s; **(c)** 50mV/s and **(d)** 100mV/s. R^2 values are shown on the plots. Red line: linear regression plot. **(e)** Plot of R^2 values of linear regression against scan rates (mV/s) of cyclic voltammetry.

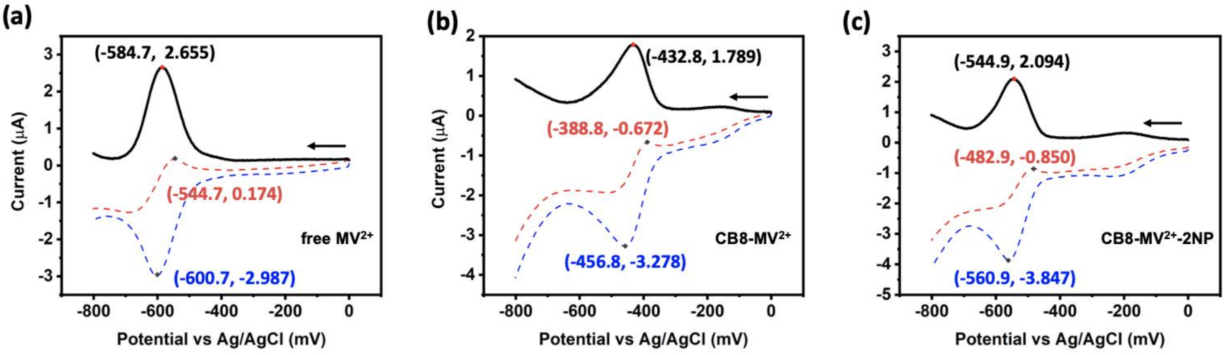

Figure S8. Overall square wave voltammetric (SWV) response (black solid line), corresponding cathodic curve (blue dash line) and anodic curve (red dash line) of **(a)** free MV^{2+} **(b)** CB8-MV^{2+} and **(c)** CB8-MV^{2+}-2NP complex. The overall SWV response plots the current difference between forward pulse ($E + E_{pulse}$) and reverse pulse ($E - E_{pulse}$) against the potential staircase, where E is applied potential (from 0 mV to -800 mV), E_{pulse} is pulse size (25 mV). The larger ΔV_{G2}, i.e. $| E_{CB8\text{-}MV\text{-}G2} - E_{CB8\text{-}MV}|$, obtained from SWV compared to that from cyclic voltammetry (CV) can be attributed to the different degrees of anodic current contribution around cathodic peak potential between CB8-MV^{2+} and CB8-MV^{2+}-G2 at each pulse of SWV. Specifically, the reduction peak of CB8-MV^{2+} measured in SWV with respect to CV positively shift more than that of CB8-MV^{2+}-G2 ternary complexes. As observed from CV (Fig. S10), splitting between cathodic peak from CB8-MV^{2+}-G2 and anodic peak from CB8-2MV^{+} is always larger than 100 mV even when scan rate is as slow as 10 mV/s, which indicates that the oxidation of kinetically stable CB8-2$MV^{+\bullet}$ requires the potential at least 100 mV more positive than the cathodic

peak potential. Since the difference in applied potential between the forward and the reverse pulses in SWV is only $2E_{pulse}$ = 50 mV, the reduced product CB8-2$MV^{+•}$ in (b) CB8-MV^{2+} is difficult to oxidize back by the reverse pulse, resulting in a supressed anodic current and a largely irreversible reaction around the cathodic peak (-456.8 mV). In the presence of G2, e.g. 2NP, the formation of CB8-2$MV^{+•}$ is suppressed and competed by the formation of CB8-$MV^{+•}$-2NP, which is less kinetically stable and can be oxidized more easily by the reverse pulse. Therefore, the anodic current around the cathodic peak potential (-560.9 mV) of CB8-MV^{2+}-2NP ternary complex is partially restored with respect to (b) CB8-MV^{2+}, i.e. the anodic peak slightly shift to a more negative value. In CV mode, the reduction potential is solely extracted from the cathodic peak, which is not affected by anodic processes. Therefore, ΔV_{G2}, i.e., $|E_{CB8\text{-}MV\text{-}G2} - E_{CB8\text{-}MV}|$, in SWV should be slightly larger than that obtained from CV.

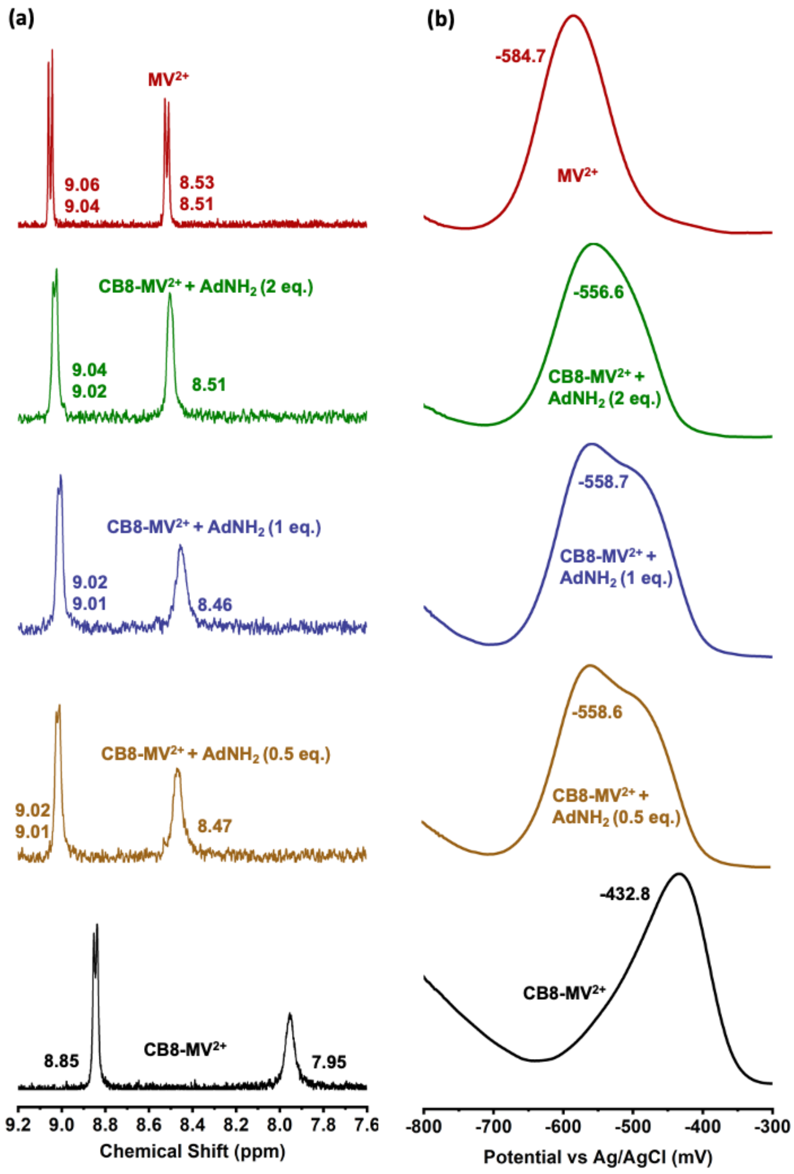

Figure S9. (a) ^{1}H NMR spectra recorded by a 400 MHz NMR equipment and **(b)** square wave voltammograms of the CB8-MV^{2+} complex in the presence of various equivalents (0, 0.5, 1 and 2) of 1-adamantylamine ($AdNH_2$), and free MV^{2+} from the bottom to the top. The convoluted peaks in SWV indicate displacement of MV^{2+} from the CB8 cavity via competitive binding of $AdNH_2$, as verified by the downfield shift of the MV^{2+} signals in the ^{1}H NMR data.

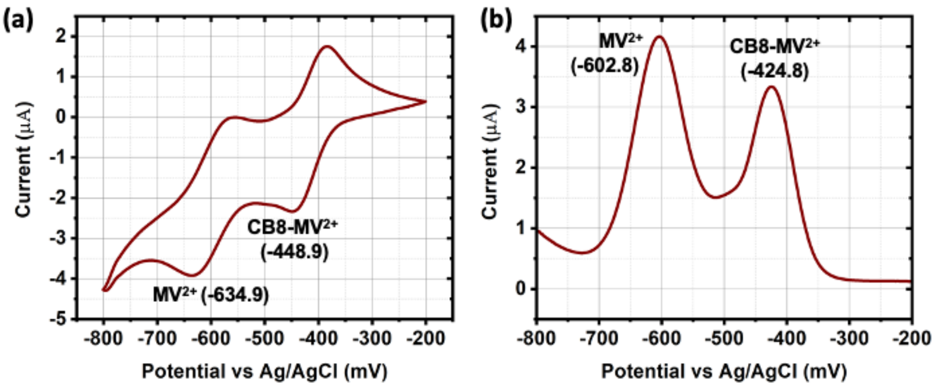

Figure S10. Electrochemical behaviors of an aqueous solution containing 1.0 mM CB8 and 2.0 mM MV^{2+} measured in **(a)** cyclic voltammetric mode at a scan rate of 10 mV/s and **(b)** square wave voltammetric mode at frequency of 5 Hz, step size of 2 mV and pulse size of 25 mV. Working electrode: gold disk (0.0314 cm^2). Reference electrode: Ag/AgCl. Medium: 6.25 mM phosphate buffer solution (pH = 7.0). Signals corresponding to both CB8-MV^{2+} and free MV^{2+} are clearly visible in CV and SWV.

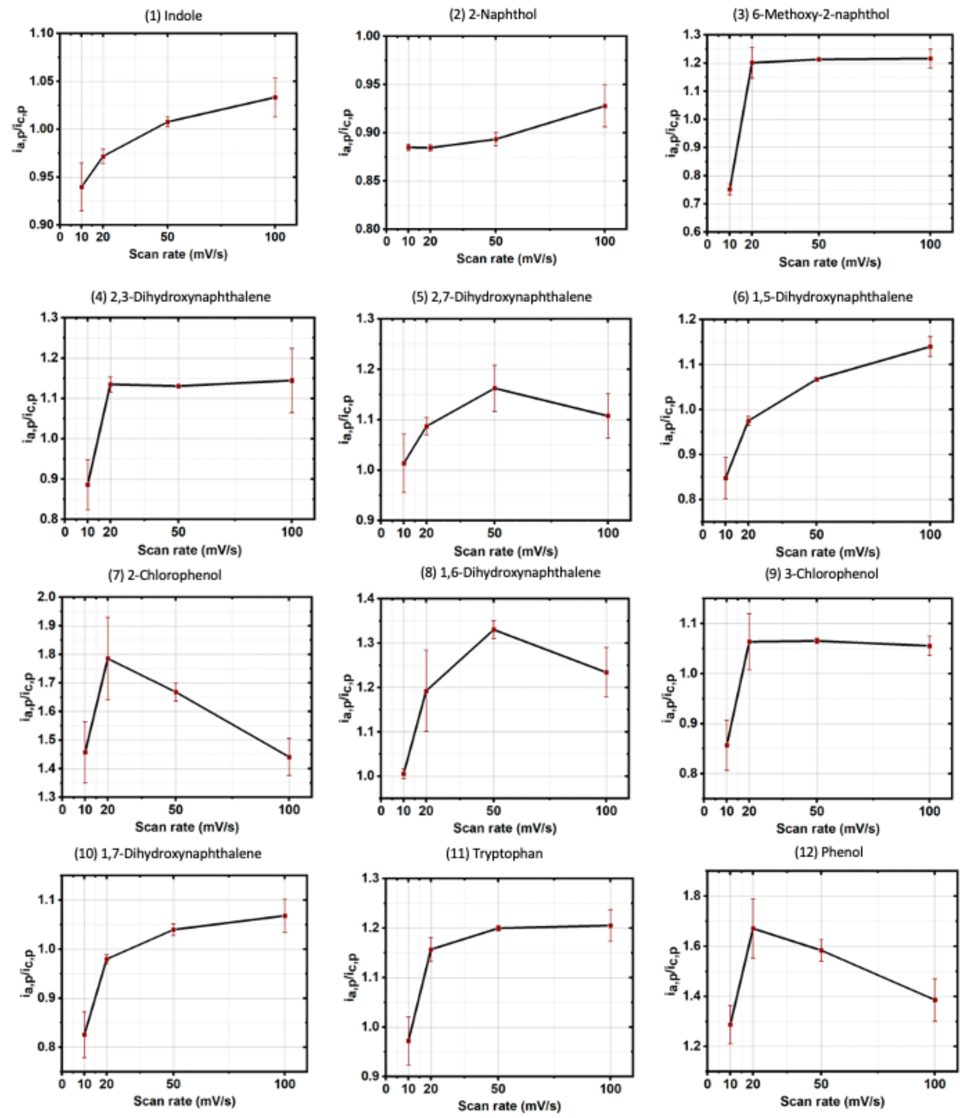

(Figure continues on page S12)

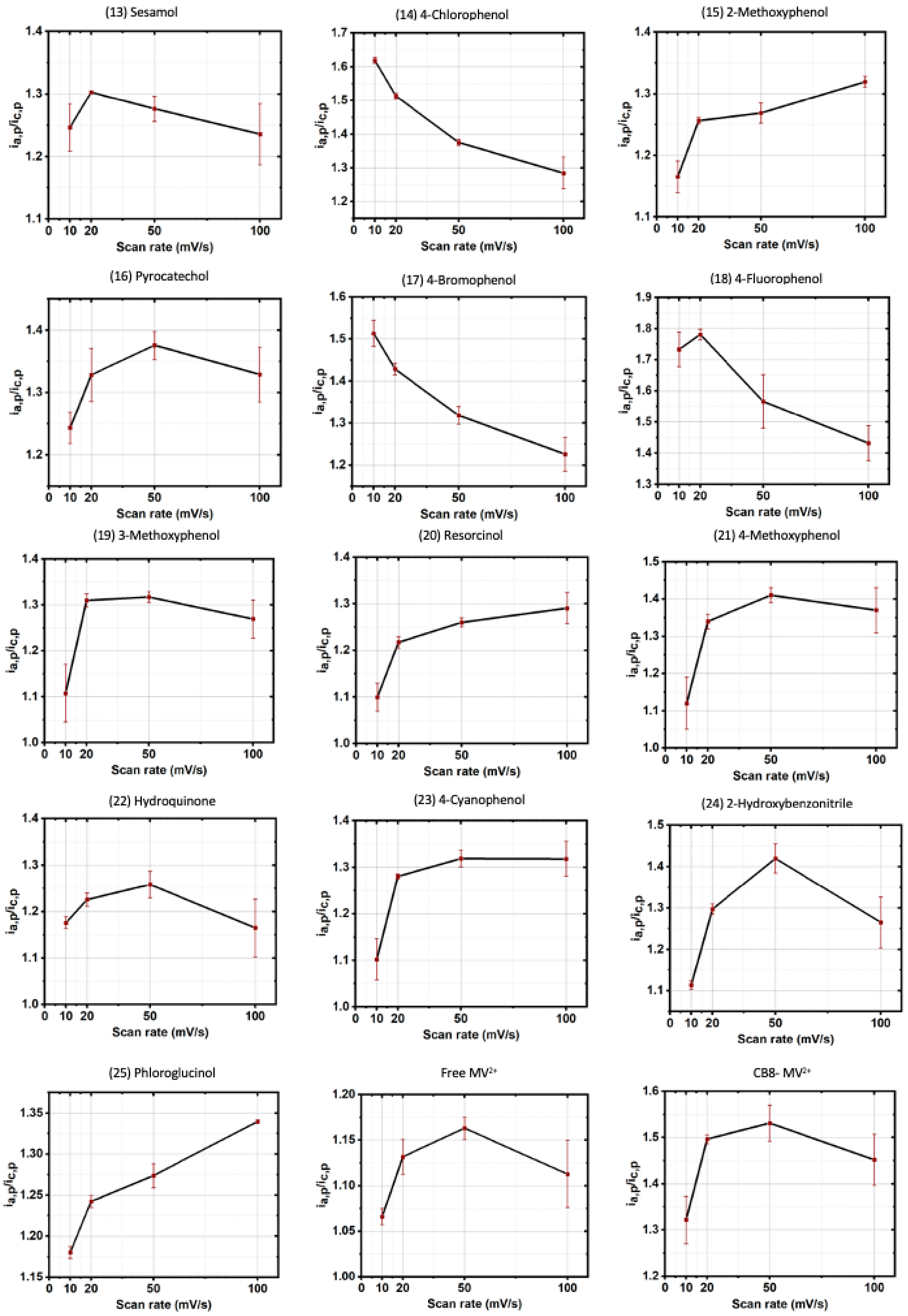

Figure S11. Peak current ratio ($i_{a,p}/i_{c,p}$) of 25 different CB8-MV^{2+}-G2 ternary complexes, free MV^{2+} and CB8-MV^{2+} at different scan rates of 10, 20, 50 and 100 mV/s. This ratio was estimated by the Nicholson method,[S1] which is given as $i_{a,p}/i_{c,p} = \left(i_{a,p}\right)_0/\left(i_{c,p}\right)_0 + 0.485 \times \left(i_{s,p}\right)_0/\left(i_{c,p}\right)_0 + 0.086$, where $i_{a,p}$ and $i_{c,p}$ are anodic and cathodic current with a proper baseline; $\left(i_{a,p}\right)_0$ and $\left(i_{c,p}\right)_0$ are

experimentally measured anodic and cathodic peak current; $\left(i_{\mathrm{s,p}}\right)_0$ is current measured at the switching potential. It is noted that the peak current ratio has a theoretical maximum value of 1. The observed larger-than-1 values come from the overestimation of the cathodic current at switching potential $\left(i_{\mathrm{s,p}}\right)_0$ in the employed Nicholson method, due to the onset of the hydrogen evolution reaction occurs at the switching potential. Error bars are the standard deviation of peak current ratio values calculated from 5 different scanning cycles in cyclic voltammetry.

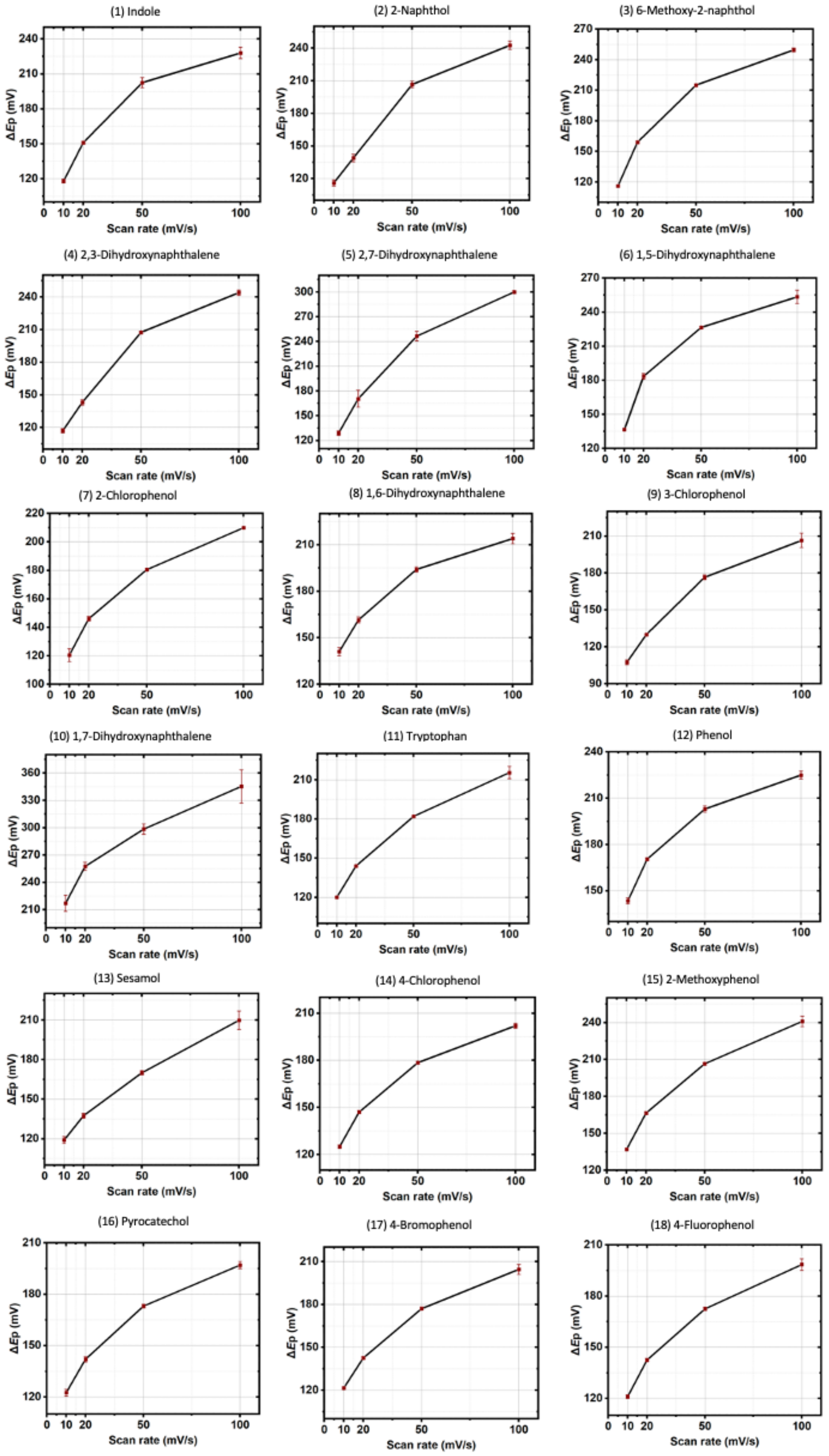

(Figure continues on page S14)

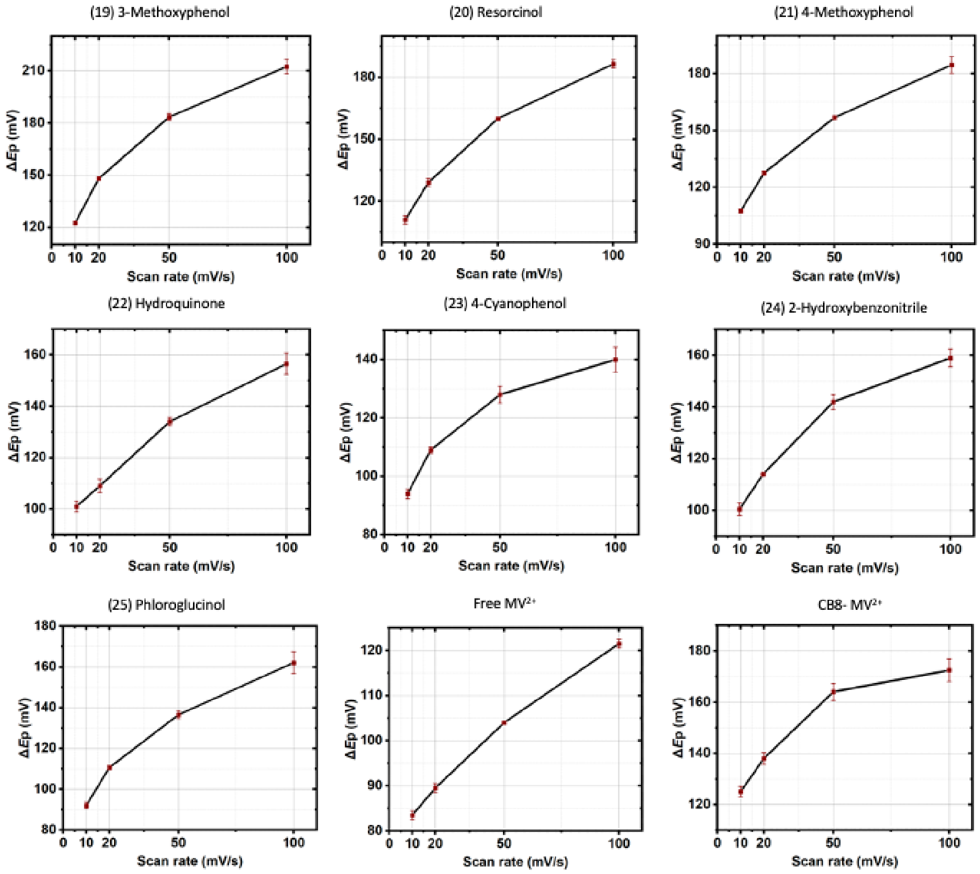

Figure S12. Peak-to-peak separation of 25 different CB8-MV^{2+}-G2 ternary complexes, free MV^{2+} and CB8-MV^{2+} at different scan rates of 10, 20, 50 and 100 mV/s. The general increase in peak-to-peak separation with increasing scan rate indicates anodic reaction in the reverse scan is significantly hindered due to the exceptional kinetic stability of the CB8-2$MV^{+\bullet}$ complex.

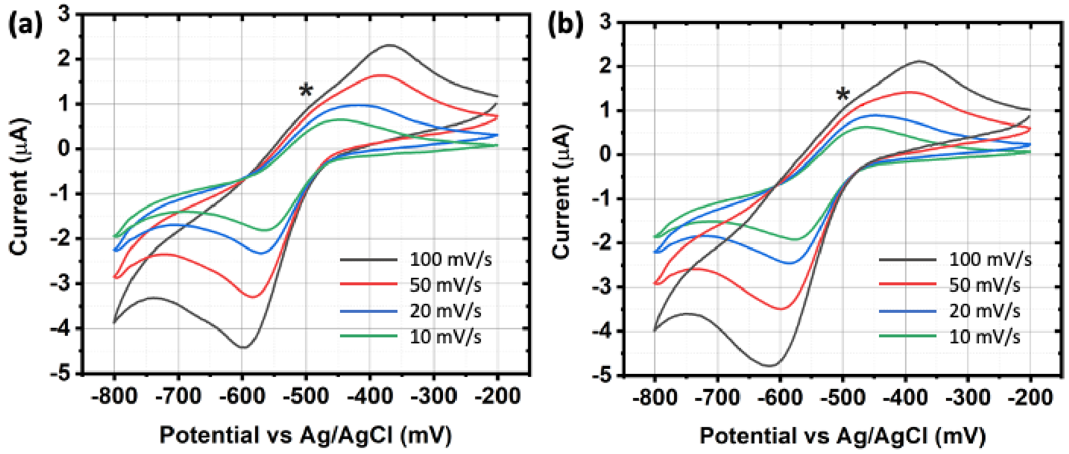

Figure S13. Cyclic voltammetric response of **(a)** CB8-MV^{2+}-indole and **(b)** CB8-MV^{2+}-2NP ternary complexes at different scan rates of 10, 20, 50 and 100 mV/s. The bulge on the negative side of the main anodic peak appeared at higher scan rates is marked by an asterisk.

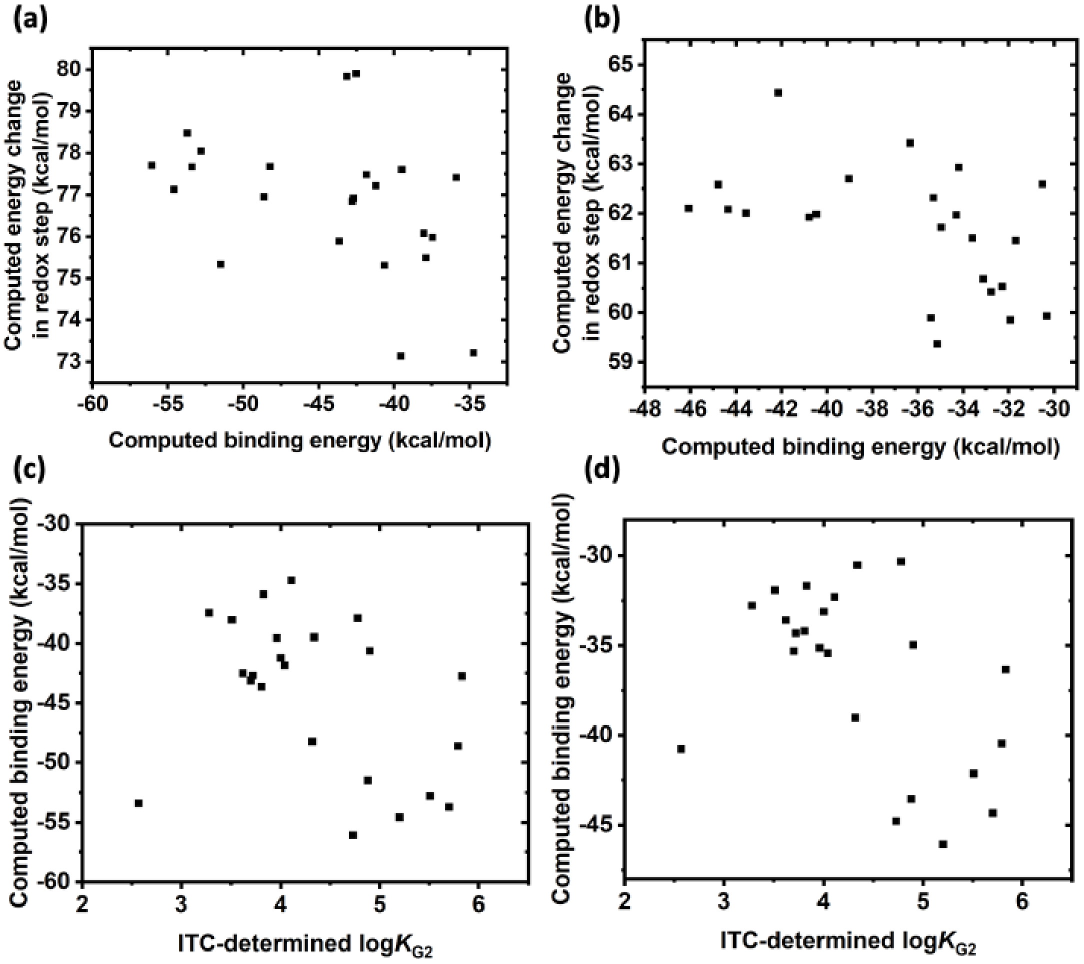

Figure S14. (a-b) Scatter plots of computed energy change in the redox step (eq. S2) against computed binding energy between CB8-MV^{2+} and G2 (eq. S1). **(c-d)** Scatter plots of computed binding energy between CB8-MV^{2+} and G2 (eq. S1) against the corresponding ITC-determined logK_{G2}. (a) and (c) were obtained at wB97XD/6-31G*; (b) and (d) were obtained at CPCM/wB97XD/6-31G* level of theory. No correlation can be observed in any of the plots. For (a) and (b), it indicates that the ΔV_{G2}-logK_{G2} correlation should be rooted in the post-reduction host-guest exchange events (eq. 2 in the main text). It is noted that eq. S2 represents the full redox reaction of the ternary complexes with the corresponding cathodic half-reaction shown as eq. 1 in the main text. Full redox reaction is considered to ensure the conservation of the number of electrons on both sides of the equation. For (c) and (d), the lack of correlation points to the important contribution of solvation energy (especially that of G2) in aqueous binding constants of the ternary complexes, which is not considered in the calculation here.

$$[\text{CB8-}MV^{2+}] + \text{G2} \rightleftharpoons [\text{CB8-}MV^{2+}\text{-G2}] \quad \text{(S1)}$$

$$[\text{CB8-}MV^{2+}\text{-G2}] + 1.5H_2O \rightleftharpoons [\text{CB8-}MV^{+\bullet}\text{-G2}] + 0.25O_2\ \text{(triplet)} + H_3O^+ \quad \text{(S2)}$$

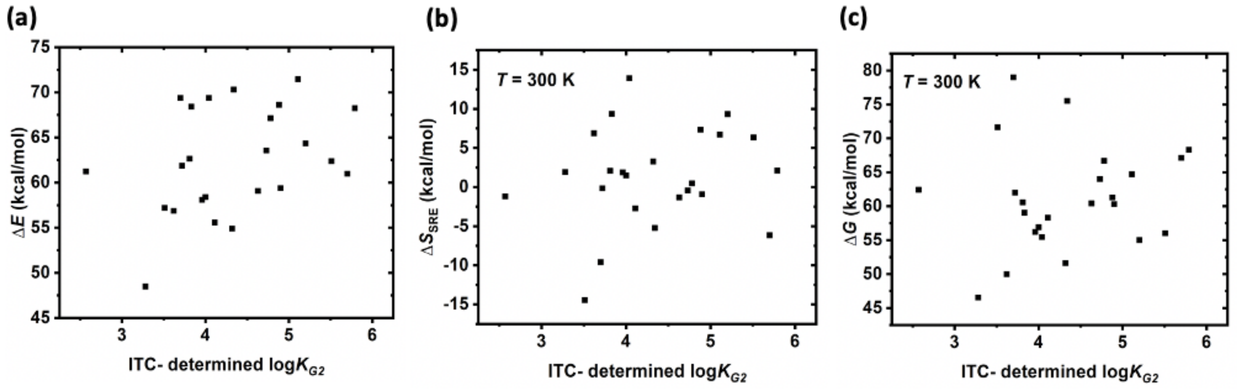

Figure S15. (a) Scatter plot of the computed energy change in the redox step (eq. S2) against the reported ITC-determined logK_{G2}. The energies of the reduced and oxidized complexes were obtained as the average energy over 30 ps trajectories at 300 K in vacuum at the PM6D3 level of theory. **(b)** Scatter plot of the configurational entropy change upon reduction of [CB8-MV^{2+}-G2] to [CB8-MV$^{+\bullet}$-G2] at 300 K obtained from the same 30 ps trajectories using the Spectrally Resolved Estimation of the entropy. **(c)** The Gibbs free energy was obtained using the computed enthalpy and entropy values (incl. translation and rotational entropy values), taking into consideration of the contribution of water oxidation as in eq. S2 to conserve the number of electrons. No correlations can be seen in the graphs with the experimental logK_{G2}.

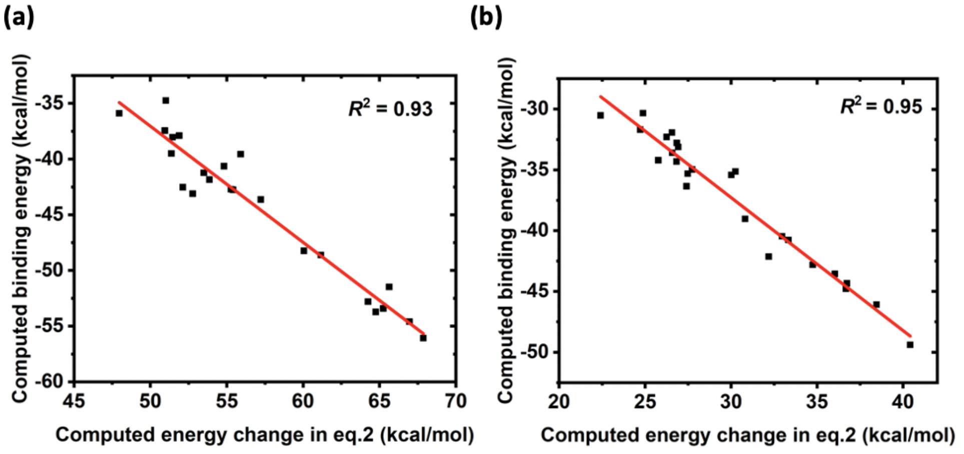

Figure S16. Correlation of the computed binding energy between CB8-MV^{2+} and G2 (eq. S1) and the computed energy change of the post-reduction host-guest exchange (eq. 2 in the main text) obtained at **(a)** wB97XD/6-31G* and **(b)** CPCM/wB97XD/6-31G* level of theory. Possible formation of CB8-G2 binary complexes will contribute to the deviation of data points from a perfect correlation. This correlation indicates that a good G2 of CB8-MV^{2+} is also a good G2 of CB8-MV$^{+\bullet}$.

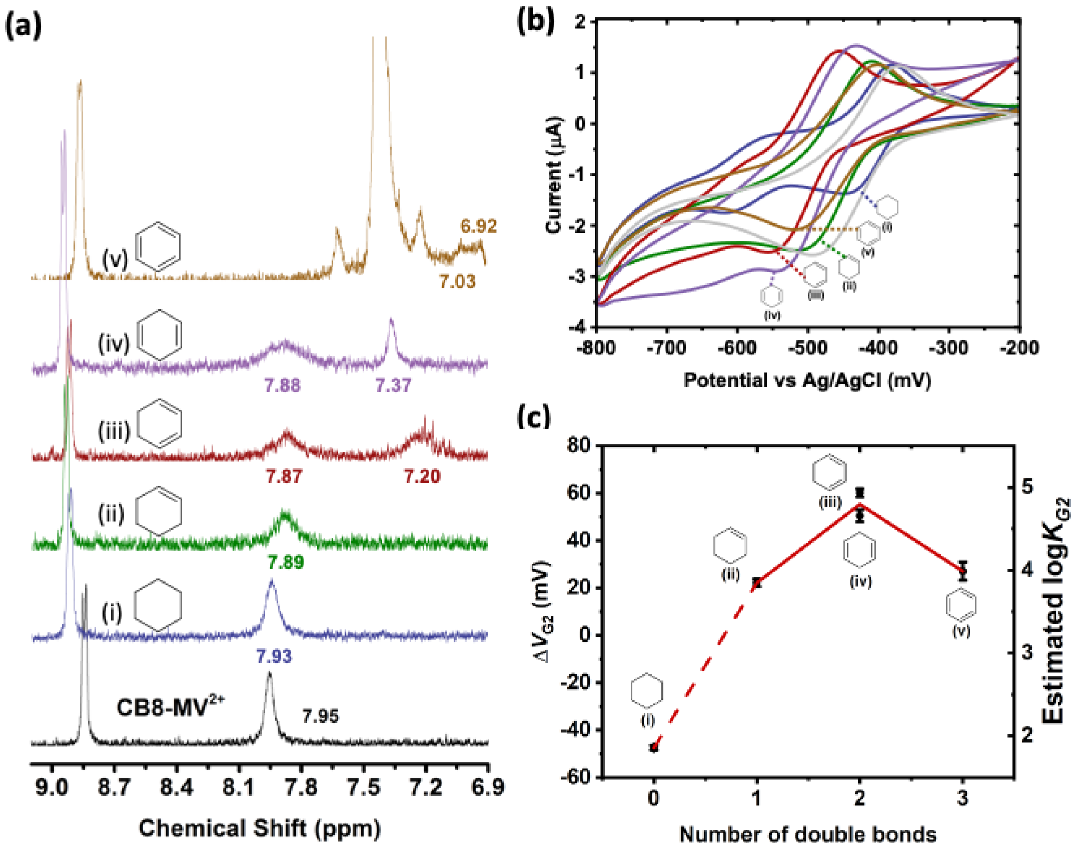

Figure S17. **(a)** ^{1}H NMR spectra recorded on a 400 MHz spectrometer and **(b)** cyclic voltammetric response on gold disk working electrode (0.0314 cm^2) of CB8-MV^{2+} (light grey line) and CB8-MV^{2+}-G2 ternary complexes. G2 = (i) cyclohexane, (ii) cyclohexene, (iii) 1,3-cyclohexadiene, (iv) 1,4-cyclohexadiene and (v) benzene; **(c)** Plot of cyclic voltammetric reduction potential shift (ΔV_{G2}) of CB8-MV^{2+}-G2 (left y-axis), and predicted binding constants ($\log K_{G2}$) of CB8-MV^{2+}-G2 complexes (right y-axis) against number of double bonds in G2.

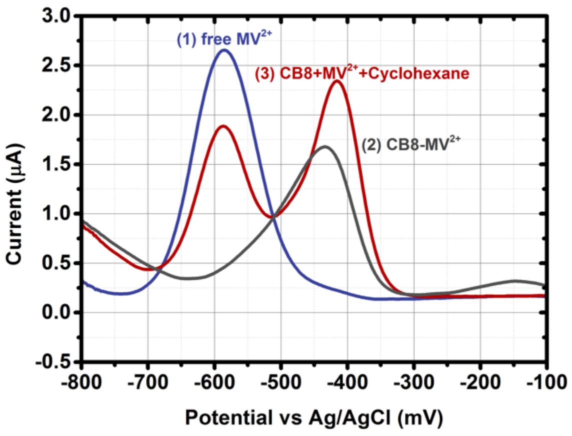

Figure S18. Square wave voltammograms of samples containing (1) 1 mM free MV^{2+}; (2) 1 mM CB8-MV^{2+}; (3) 1 mM 1:1:1 of CB8, MV^{2+} and cyclohexane. Frequency: 5 Hz. Step size: 2 mV. Pulse Size E: 25 mV. Working electrode: gold disk (0.0314 cm^2). Reference electrode: Ag/AgCl. Medium: 6.25 mM phosphate buffer solution (pH = 7.0).

2. References

(S1) Nicholson, R. S. Semiempirical Procedure for Measuring with Stationary Electrode Polarography Rates of Chemical Reactions Involving the Product of Electron Transfer. *Anal. Chem.* 1966, *38*, 1406-1406.

(S2) Rauwald, U.; Biedermann, F.; Deroo, S.; Robinson, C. V.; Scherman, O. A. Correlating solution binding and ESI-MS stabilities by incorporating solvation effects in a confined cucurbit [8] uril system. *J. Phys. Chem. B* 2010, *114*, 8606-8615.
